# Mechanisms of the Oxygen Evolution Reaction on NiFe_2_O_4_ and CoFe_2_O_4_ Inverse-Spinel Oxides

**DOI:** 10.1021/acscatal.2c01534

**Published:** 2022-07-13

**Authors:** Öyküm N. Avcı, Luca Sementa, Alessandro Fortunelli

**Affiliations:** † CNR-ICCOM, Consiglio Nazionale delle Ricerche, Via G. Moruzzi 1, Pisa 56124, Italy; ‡ Department of Chemistry and Industrial Chemistry, DSCM, University of Pisa, Via G. Moruzzi 13, Pisa 56124, Italy; § CNR- IPCF, Istituto per i Processi Chimico-Fisici, Via G. Moruzzi 1, Pisa 56124, Italy

**Keywords:** oxygen evolution reaction, DFT, reaction mechanism, electrocatalysis, spinel oxides, Ni−Fe oxides, Co−Fe oxides

## Abstract

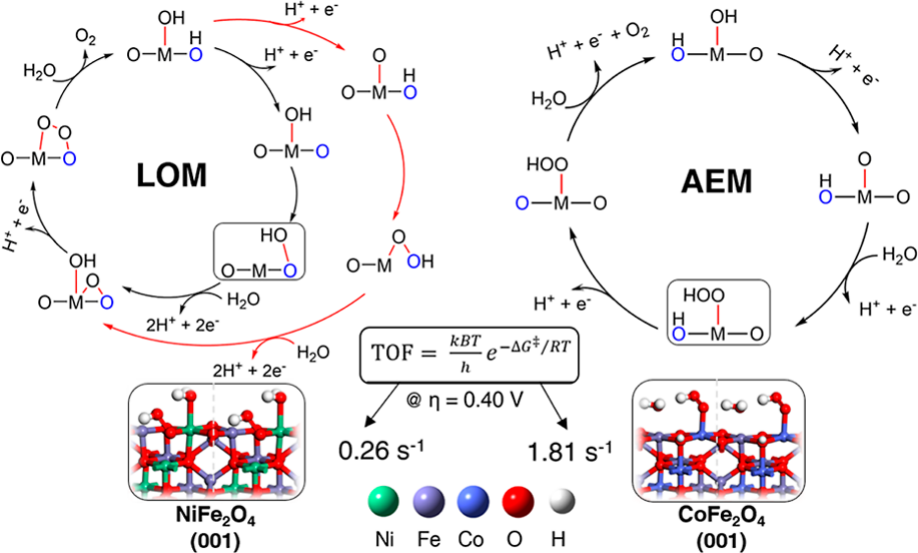

Spinel ferrites, especially Nickel ferrite, NiFe_2_O_4_, and Cobalt ferrite, CoFe_2_O_4_, are efficient and promising anode catalyst materials in the field of electrochemical water splitting. Using density functional theory, we extensively investigate and quantitatively model the mechanism and energetics of the oxygen evolution reaction (OER) on the (001) facets of their inverse-spinel structure, thought as the most abundant orientations under reaction conditions. We catalogue a wide set of intermediates and mechanistic pathways, including the lattice oxygen mechanism (LOM) and adsorbate evolution mechanism (AEM), along with critical (rate-determining) O–O bond formation barriers and transition-state structures. In the case of NiFe_2_O_4_, we predict a Fe-site-assisted LOM pathway as the preferred OER mechanism, with a barrier (Δ*G*^⧧^) of 0.84 eV at *U* = 1.63 V versus SHE and a turnover frequency (TOF) of 0.26 s^–1^ at 0.40 V overpotential. In the case of CoFe_2_O_4_, we find that a Fe-site-assisted LOM pathway (Δ*G*^⧧^ = 0.79 eV at *U* = 1.63 V *vs* SHE, TOF = 1.81 s^–1^ at 0.40 V overpotential) and a Co-site-assisted AEM pathway (Δ*G*^⧧^ = 0.79 eV at bias > *U* = 1.34 V *vs* SHE, TOF = 1.81 s^–1^ at bias >1.34 V) could both play a role, suggesting a coexistence of active sites, in keeping with experimental observations. The computationally predicted turnover frequencies exhibit a fair agreement with experimentally reported data and suggest CoFe_2_O_4_ as a more promising OER catalyst than NiFe_2_O_**4**_ in the pristine case, especially for the Co-site-assisted OER pathway, and may offer a basis for further progress and optimization.

## 1 Introduction

Water electrolysis is a well-established method to produce hydrogen from renewable energy sources,^1−4^ composed of the oxygen evolution reaction (OER) and the hydrogen evolution reaction as the two half reactions involved at the anode and cathode, respectively. The kinetics of the electrochemical water splitting process is greatly hindered by the sluggish anodic OER. The OER is challenging because a difficult oxygen–oxygen bond formation step in addition to four proton-coupled electron transfers under acidic conditions (2H_2_O → O_2_ + 4H^+^ + 4e^–^) or four hydroxyl-coupled electron transfers under basic conditions (4OH^–^ → O_2_ + 2H_2_O + 4e^–^) are involved. Owing to both difficulties in the oxygen–oxygen bond formation step and because multiple electron transfer is not kinetically favored, the OER is still in need of efficient catalysts to accelerate the reaction and lower the kinetic overpotential. Despite many decades of intensive research, in fact, a sizeable overpotential is observed even on the most active, state-of-the-art precious-metal catalysts, Ru and Ir and their oxides, which otherwise work efficiently under both acidic and alkaline conditions.^5−8^ Moreover, since the use of precious metals is not sustainable in world-scale applications due to their high cost and scarcity, research is currently focusing on non-noble metals, that is, Fe, Ni, Co, Cu, or other 3d transition metals. Some of these systems are indeed promising and active OER catalysts. For example, Ni-, Fe-, and Co-based oxides are chemically stable in alkaline media, and they show an OER performance not far from that of the oxides of Ru and Ir.^9−11^ Furthermore, combinations of these metals into bimetallic systems have demonstrated to highly increase the catalytic activity and stability for this electrochemical reaction.^12^ Especially layered oxyhydroxides^13−17^ and spinel-type oxides^18−21^ have attracted significant attention, and many studies have appeared investigating their chemical properties, synthetic methodologies, and catalytic performance.

**Figure 1:**
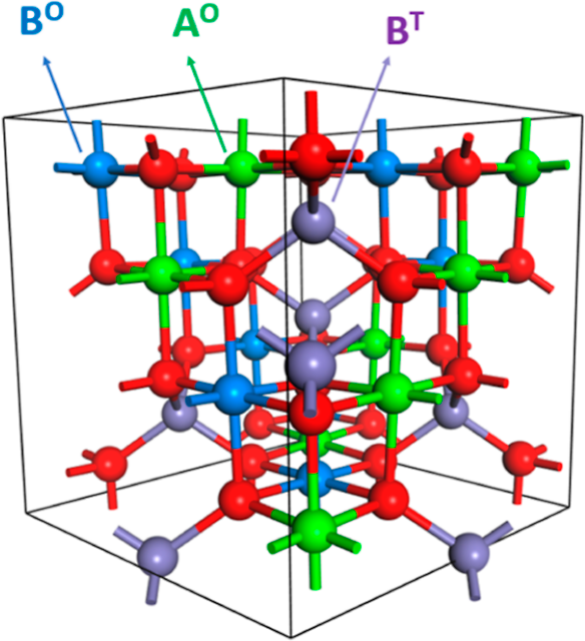
Bulk model (*n*. AB_2_O_4_) for inverse spinel oxide in ball and stick representation. O and T denote octahedral and tetrahedral coordination of A and B metals, respectively.

In particular, spinel-like structures are considered promising OER electrocatalysts due to their high electrical conductivity, structural stability, and catalytic performance, stemming from the multiple valences of the cations and the ability to switch among different oxidation states.^22^ The general formula of spinel-type oxides is (AB_2_O_4_) and consists of a cation A (M^+2^) that occupy tetrahedral sites and a cation B (M^+3^) that occupy octahedral sites of the close-packed cubic Fd3̅m[227] structure. Depending on which cations occupy octahedral or tetrahedral sites, the spinel is named as either a normal or inverse spinel structure. If A^+2^ cations occupy tetrahedral sites and B^+3^ cations occupy only octahedral sites, the structure is called “normal spinel,” whereas in the inverse spinel structure A^+2^ cations occupy half of the octahedral sites, while half of the B^+3^ cations are in tetrahedral sites and the other half occupy octahedral sites (Figure 1).

Among spinel oxide structures, spinel ferrites are of particular interest because of their low cost, high catalytic activity, and durability at high pH.^18,22^ Fe is considered the active OER catalytic species/site, but synergistic effects are observed when Fe is combined with other metals. In earlier studies, it was reported that addition of iron is beneficial for lowering the overpotential of a nickel oxide electrode,^23^ therefore, strongly promoting the OER.^24,25^ Significantly, Singh et al. noted that the NiFe bimetallic compound (spinel NiFe_2_O_4_) outperforms pure Fe (spinel Fe_3_O_4_) and Ni (cubic NiO) oxides in OER electrocatalytic activity.^26^ Various successive theoretical studies predicted that controlled Fe doping can reduce the overpotential.^27,28^ Li and Selloni^27^ performed a theoretical analysis of key configurations of pure and Fe-doped NiO_*x*_ systems and suggested that the OER activity can be enhanced when Fe doping is in the range of low to moderate level, and that NiFe_2_O_4_ is a promising OER catalyst among the studied Fe-doped NiO_*x*_. Xiao *et al.*(28) emphasized that in addition to high spin Fe stabilizing the O radical in the in (Ni, Fe) OOH systems, closed shell d^6^ Ni(IV) plays an important role in catalyzing the O–O coupling. At the experimental level, by means of spectroscopic characterization, Landon *et al.* reported that Fe-doped NiO_*x*_ contains the spinel NiFe_2_O_4_ phase and proposed that this NiFe_2_O_4_ phase is responsible for improving the OER activity of the mixed metal oxide systems.^29^

Among the wide variety of spinel ferrites (MFe_2_O_4_, M = Co, Ni, Mn, Cu *etc.*), several 3d metals have been investigated in addition to Ni. Especially cobalt ferrite (*i.e.*, CoFe_2_O_4_) is also in focus as a promising OER catalyst. Si *et al.* reported that CoFe_2_O_4_ and NiFe_2_O_4_ exhibited a higher OER activity than MnFe_2_O_4_ among prepared mesoporous nanostructured spinel ferrites,^30^ in good agreement with the trends reported by Li *et al.*(31) The cobalt/iron synergy in the OER is, therefore, worth investigating, although so far, it has not been explored as much as the synergistic effect of Ni–Fe. In particular, computational modelling could help determining the active sites of Co/Fe oxides, a point which is not fully clear at the experimental level. Indeed, by means of (spectro) electrochemical experiments including EXAFS and XAS Smith *et al.* reported the coexistence of multiple electrocatalytic sites in a series of iron-cobalt oxides with different compositions and suggested that Fe can act both directly and indirectly as an OER catalyst in this series but were not able to provide a full clarification.^32^ As we will see below, our calculations indeed suggest that Co and Fe sites could play a synergistic role, and there might be coexistence of active sites in CoFe_2_O_4_. In terms of pure Co oxide OER catalysts (*e.g.*, Co_3_O_4_, CoOOH), experimental evidence suggests for two site mechanisms containing two Co centers.^33,34^

At variance with the numerous experimental investigations, theoretical mechanistic studies on OER spinel oxide catalysts are still scarce, despite the fact that an atomistic understanding of the target reaction mechanisms is potentially very useful and possibly decisive to designing electrocatalysts that optimize OER activity and selectivity. Our aim here is to fill this gap by investigating in detail through a DFT approach, the OER reaction mechanisms on inverse-spinel ferrite catalyst NiFe_2_O_4_ and CoFe_2_O_4_ selected (001) facet. We start with NiFe_2_O_4_ (001), on which a previous theoretical study exists,^27^ and we make progress with respect to existing knowledge by: (i) investigating in detail the mechanisms of the O–O bond formation process *via* the alternative paths of lattice oxygen mechanism (LOM)^35−39^ and adsorbate evolution mechanism (AEM),^40−42^ and (ii) considering a much larger set of reaction intermediates so as to ensure that we explore all possible reaction paths. This allows us to determine a complete reaction free-energy diagram, thus providing the basis for a comparison with experimental kinetics. In addition, we conduct an analogous study by bringing another promising spinel, CoFe_2_O_4_, into our focus. We underline that, in the previous theoretical literature on MFe_2_O_4_ systems for the OER of NiFe_2_O_4_, only a limited number of local minimum intermediates were reported, and the O–O bond formation mechanism was not investigated. The lack of mechanistic research is even more severe for CoFe_2_O_4,_ for which—to the best of our knowledge—no detailed reaction free-energy profile, and thus, no mechanism has been investigated so far. To the best of our knowledge, the present study is then the first in which the O–O bond formation barrier with transition-state (TS) structures on NiFe_2_O_4_ and CoFe_2_O_4_ inverse-spinel surfaces for the OER is illustrated and quantitatively modeled. Our calculations show that on NiFe_2_O_4_ (001), a Fe-site-assisted mechanism is strongly preferred *via* a LOM pathway and presents a barrier (Δ*G*^⧧^) of the rate-determining-step (rds) occurring for the O–O bond formation of Δ*G*^⧧^ = 0.84 eV at *U* = 1.63 V versus standard hydrogen electrode (SHE), whence a computationally predicted turnover frequency (TOF) of 0.26 s^–1^ at 400 mV overpotential. As for CoFe_2_O_4_ (001), we find that Co and Fe sites could play a synergistic role, and there might be multiple active sites in agreement with experiment. In the Co-site-assisted mechanism, an AEM pathway is favored with the barrier (Δ*G*^⧧^ = 0.79 eV at bias > *U* = 1.34 V *vs* SHE) corresponding to a TOF of 1.81 s^–1^ at bias >1.34 V, while in the Fe-site-assisted mechanism, a LOM pathway is favored with the barrier Δ*G*^⧧^ = 0.79 eV at *U* = 1.63 V versus SHE, whence a TOF of 1.81 s^–1^ at 400 mV overpotential. The computationally predicted turnover frequencies exhibit good agreement with experimentally reported values. Our results also suggest that CoFe_2_O_4_ is a more promising OER catalyst than NiFe_2_O_**4**_ in pristine case, especially in the Co-site-assisted OER mechanism.

## 2 Computational Approach

Spin-polarized density functional theory plus Hubbard correction (DFT + *U*) calculations were performed in the plane wave and ultrasoft pseudopotential framework,^43^ as implemented in the Quantum-Espresso suite of codes.^44^ The PBE^45^ exchange–correlation functional was used in the DFT part, augmented with Hubbard U parameters chosen as 3.3, 5.5, and 4.5 eV for Fe, Ni, and Co, respectively.^27,46^ Kinetic energy cutoffs of 40 and 200 Ry were chosen for describing the wave function and the charge density, respectively. Nudged elastic band (NEB) calculations,^47^ including climbing image (CI),^48^ were used to find reaction barriers and transition states.

**Figure 2:**
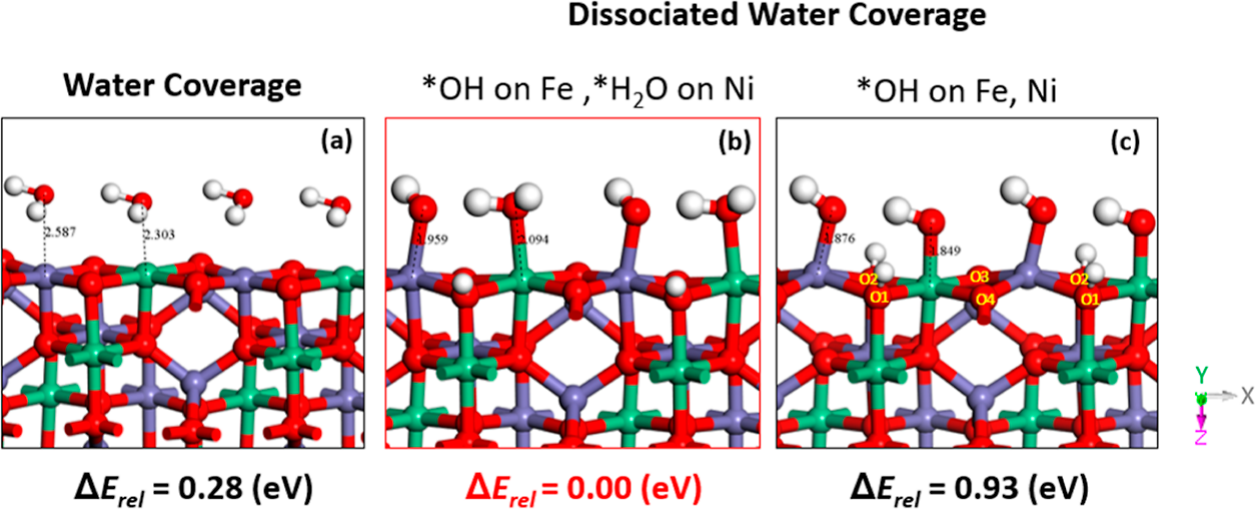
Coverage patterns on NiFe_2_O_4_ (001). (a) Surface covered with adsorbed undissociated water molecules on each metal sites and nonprotonated lattice oxygens. (b) Adsorbed OH (*OH) on the Fe site, while water is still adsorbed (*H_2_O) on Ni site, and one lattice surface oxygen is protonated. (c) Adsorbed *OH on both Ni and Fe metal sites, while two lattice surface oxygens are protonated. Oxygen, hydrogen, iron, and nickel atoms are colored red, white, violet, and green, respectively.

The selected catalysts NiFe_2_O_4_ and CoFe_2_O_4_ inverse spinel structures are ferrimagnetic,^49,50^ that is, their ferrimagnetic spin arrangement is ↑↓↑ for M1(Oh)/Fe(Td)/Fe(Oh), (M1 = Ni, Co), respectively, where we use the notation: spin up (↑) and spin down (↓). We indeed found these spin arrangements as a stable state in our calculations. Perron *et al.* reported computationally that an inverse ferrimagnetic arrangement for NiFe_2_O_4_ is the most stable state amongst various calculated spin arrangements.^49^ Hossain *et al.*(51) reported that CoFe_2_O_4_ is also highly spin-polarized inverse spinel and stated that the exchange interaction between the up and down spin states in the octahedral and tetrahedral regions due to crystal field effects results in a high spin. Caffrey *et al.*(52) assessed the potential of the ferrimagnetic spinel ferrites NiFe_2_O_4_ and CoFe_2_O_4_ to act as spin-filtering barriers in magnetic tunnel junctions in agreement with previous calculations. The magnetic moments (μB) of the metal atoms in the bulk were calculated as follows: 1.65, 4.19, −4.11 for Ni, Fe (Oh), Fe(Td), respectively, for NiFe_2_O_4_; and 2.67, 4.17, −4.10 for Co, Fe (Oh), Fe(Td), respectively, for CoFe_2_O_4_. The magnetization of the octahedral Ni (1.65 μB) can be assigned to the two unpaired electrons of a low-spin t_2g_^6^ e_g_^2^ configuration, in agreement with its divalent nature (Ni^2+^, d^8^). The magnetization of octahedral Co (2.67 μB) can be assigned to the three unpaired electrons of a high-spin t_2g_^5^ e_g_^2^ configuration, in agreement with its divalent nature (Co^2+^, d^7^). The Fe ions in the octahedral and tetrahedral positions have a similar magnetization of about 4.2 μB, which can be assigned to the approximately five unpaired electrons of a high-spin configuration, t_2g_^3^ e_g_^2^ (Oh site) and e^2^ t_2_^3^ (Td site), in agreement with its trivalent nature (Fe^3+^, d^5^). Note that since we predict that both spinels are ferrimagnetic in their ground states, the magnetic moments of the Oh and Td regions are opposite in sign. We focus in both cases on the (001) surface, that is, one of the most frequently exposed surface in the spinel structures.^27,53−55^ Symmetric, nonstoichiometric slabs were utilized, 15 layers thick, corresponding to a total of 55 atoms per unit cell (see the Supporting Information, Figure S1), similarly to previous work.^27^ To clarify the nomenclature of surface states/configurations employed in the following, we note that our top slab surfaces exposed to adsorbates exhibit 2 metal sites and 4 oxygens, see Figure S1. The 4 oxygens are labeled as O1, O2, O3, and O4 in Figure 2c and also in Figure S1. In addition to the full set of intermediates for LOM and AEM pathways shown in this article, in the Supporting Information Figures S14–S16, we also denote coverage using the following notation: [adsorbate on Fe/adsorbate on O1 and (or) O2\adsorbate on Ni or Co], for example, OH/O1H\H_2_O indicates that top Fe sites carry OH (*OH), O1 sites are protonated, and the Ni (or Co) sites are covered with water. A 3 × 3 × 1 Monkhorst-Pack *k*-point mesh was utilized for energy and structural calculations. Slab surface unit cells with dimensions of 5.89 Å × 5.89 Å × 32 Å for NiFe_2_O_4_ and 5.93 Å × 5.93 Å × 32 Å for CoFe_2_O_4_ were built using experimental bulk-cubic lattice parameters.^56^

To model the thermochemistry of the OER, it is convenient to work under acidic conditions because charged intermediates under basic conditions make it difficult to carry out DFT calculations and require tremendous time and effort.^57,58^ Noting that the OER reaction (2H_2_O → O_2_ + 4H^+^ + 4e^–^) can be rewritten as (4OH^–^ → 2H_2_O + O_2_ + 4e^–^) under a basic (alkaline) environment, substitution of the water dissociation equilibrium H_2_O (l) ⇌ H^+^ (aq) + OH^–^ (aq) into elemental steps naturally allows one to convert the results under acidic into basic conditions.^59^ Both are equivalent from the thermodynamic perspective. However, the reacting species are different in the two cases, and the kinetic barriers may be affected by the difference. To explore how this may change the mechanism and its energetics, we thus performed charged and implicit solvent calculations for one step (*O + OH^–^ → *OOH + e^–^) of the OER reaction under alkaline conditions on CoFe_2_O_4_ (see the Supporting Information for detail).

In the reactions involving gaseous or liquid molecules such as oxygen, hydrogen, and water, it is essential to include entropy terms when obtaining the system free-energy (*G*). In our work, we derived the OER free energies using the same scheme as utilized in previous studies.^27,60a^ The total reaction energy (Δ*E*) of each elementary step was obtained directly *via* our DFT calculations. The contributions (Δ*H*, ΔZPE, and *T*Δ*S*) to the free energies (Δ*G*) for small molecules (H_2_O and H_2_) were added empirically from previous studies^27,60a^ as follows: Δ*G*_i_ = Δ*E*_i_ + Δ*H*_i_ + ΔZPE_i_ – *T*Δ*S*_i_ (see the Supporting Information, Table S1), which was reported that Li *et al.*(60a) utilized standard thermodynamic data^60b^ to obtain the T and p contributions to the G values of aqueous H_2_O and gaseous H_2_. The free energy of O_2_ is expressed as *G*[O_2_] = 4.92 eV + 2*G*[H_2_O] – 2*G*[H_2_] according to the OER equilibrium under standard conditions because calculation of the O_2_ molecule bond energy is difficult to determine accurately using GGA-DFT, as proposed in previous work.^58^

We considered the SHE as a reference (standard conditions: pH = 0, p = 1 bar, *T* = 298.15 K), so the proton {*G*[H^+^]} and electron {*G*[e^–^]} free energies are replaced by 1/2 *G*[H_2_] according to the following eq 1
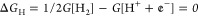
1

Since the electron–hole pair generation occurs in electrocatalytic processes, it is more convenient to rewrite an elementary step involving protons and holes in eq 2 as eq 3
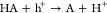
2
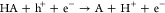
3

Then, using the SHE as the reference as implicit in eq 1, we can write the free energy change in elementary steps as eq 4
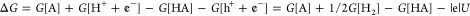
4where *G*[H_2_] is the free energy of H_2_ in the gas phase under standard conditions, and *U* is the electrode potential versus the SHE, and thus |e|*U* represents the energy required to generate the electron–hole pair.

## 3 Results and Discussion

Previous literature modelling OER on the catalytic systems investigated in the present work was limited to few intermediate structures and did not consider associated energy barriers. Disregarding barriers in the OER path is reasonable for the OER steps involving simple deprotonation processes associated with electrochemical oxidation, which can be assumed to be fast, as they typically present barriers of few kcal/mol, as observed in similar Grotthus-like mechanisms of proton transfer in aqueous electrolytes.^61,62^ This is, however, not justified for the key catalytic step before the final O_2_ evolution and, in particular, the O–O coupling (oxygen-oxygen bond formation) that is known to exhibit very often a significant barrier.^28^ Here, in addition to illustrating this key O–O bond formation step, we also explored several coverage patterns in order to determine an overall OER reaction path and to investigate more thoroughly the associated OER catalytic cycle on [Ni, Co]Fe_2_O_4_ spinel oxides. We focus in both cases on the (001) surface, that is, the most frequently exposed surface in the spinel structures/nanoparticles,^53−55^ and has less vacancy in surface metal sites on octahedral positions that are filled with adsorbates (OH, OH_2_, and OOH). We use the electrochemistry model developed by Nørskov *et al.*(59) for electrochemical systems to calculate how the relative energies of intermediates depend on the bias *U* and apply this approximation to the proton electron (H^+^ + e^–^) transfers steps (shown in eq 4), considering that the overall charge of the system is constant. For definitiveness, we work at an applied bias of *U* = 1.48 V versus SHE to deduce the reaction free energies, as this bias is at present the optimal/realistic target of only 0.25 V overpotential and is considered as the one realistically closest to the thermos-neutral voltage for water electrolysis (1.23 V).

### 3.1 Coverage Patterns

To begin with, undissociated waters were included to cover and fill coordination of each metal site on the (001) surface. Then, coverage by hydroxyls obtained *via* water dissociation (*OH + *H, and then in a later stage by oxo-groups: *OH → *O + *H) was considered, and the relative affinity of *H_2_O, *OH, and *O on the metal sites were predicted by calculating the relative energy of the configurations associated with each coverage pattern. The sampling of several coverage patterns on the catalyst surface led us to determine the lowest free-energy OER pathway.

#### 3.1.1 NiFe_2_O_4_ and CoFe_2_O_4_

In Figure 2, we depict the NiFe_2_O_4_ (001) with three different coverage options on the metal sites, together with their relative energetics: (1) adsorbed undissociated waters on each metal sites, (2) adsorbed OH (*OH) on Fe sites, one protonated lattice surface oxygen and adsorbed (*H_2_O) on Ni sites; (3) adsorbed *OH on both Ni and Fe metal sites and two protonated lattice surface oxygens. Note that the three configurations in Figure 2 have the same overall stoichiometry; thus, the total electronic energies can be directly compared. Note also that, for case (2), the alternative option (*OH on Ni and *H_2_O on Fe) has been also tried; however, it eventually converged to that illustrated in Figure 2b, which results as the lowest energetically and thus the favored configuration to initiate OER reaction on this catalyst. We can conclude that, while Ni sites mostly prefer *H_2_O adsorption, Fe sites prefer *OH adsorption as stable resting-state adsorbates. This also suggests that Fe cations are the active sites for the OER reaction to take place, as widely reported in experimental studies.^63^ Full water dissociation into adsorbed OH on both metal cations with protonated surface oxygen anions (O1 and O2), as shown in Figure 2c, appears to be the least favorable state to initiate the catalytic cycle considering its 0.93 eV higher energy. We note that the neighboring lattice oxygens (O3, O4) are coordinated to the tetrahedral Fe atom in the second layer (see Figure S2 for a side view), and we found that their protonation implies higher energies: it thus seems that coordination of oxygens with tetrahedral metals is not favorable to protonation, possibly because of the coordination angle and its steric reasons or for crystal-field orbital-geometry reasons. We recall, in fact, that crystal field splitting is smaller in the tetrahedral field compared to the octahedral field, and a lesser number of ligands are involved. Moreover, in the tetrahedral crystal field, the d-orbitals (t_2_, e) do not point directly toward the ligands to reduce electron–electron repulsion, and this can make the tetrahedral field energetically unfavorable to receive electron pairs.

**Figure 3:**
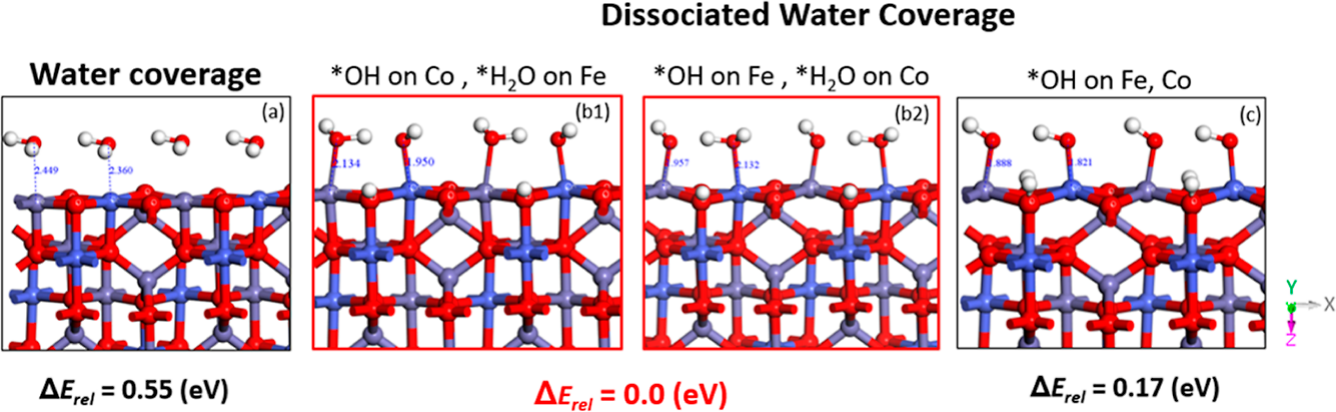
Coverage patterns on CoFe_2_O_4_ (001). (a) Surface covered with adsorbed undissociated water molecules on each metal sites and nonprotonated lattice oxygens. (b1) Adsorbed OH (*OH) on the Co site, while water is still adsorbed (*H_2_O) on the Fe site, and one lattice surface oxygen is protonated. (b2) Adsorbed OH (*OH) on the Fe site, while water is still adsorbed (*H_2_O) on the Co site, and one lattice surface oxygen is protonated. (c) Adsorbed *OH on both Fe and Co metal sites, while two lattice surface oxygens are protonated. Oxygen, hydrogen, iron, and cobalt atoms are colored red, white, violet, and indigo blue, respectively.

As for CoFe_2_O_4_ of (001), we followed a similar protocol with first considering full water coverage (Figure 3a) and then one-by-one dissociated patterns. Similarly to NiFe_2_O_4_, dissociated water coverage patterns on the CoFe_2_O_4_ surface are the most favorable; however, in this case, both alternatives of one-degree dissociation of water (*i.e.*, *OH on M1, *H_2_O on M2, and *vice versa*) resulted as lowest energy states [see Figure 3b1, b2]. Another difference of the CoFe_2_O_4_ surface is that the *OH coverage (Figure 3c) is more stable than *H_2_O coverage (Figure 3a), in stark contrast to NiFe_2_O_4_. Considering also that for CoFe_2_O_4_, one-degree dissociation states [Figure 3b1,b2] are equally stable on the two metal sites, this might explain why synergistic effects on Co–Fe are stronger than Ni–Fe. Co might be active as much as Fe in this catalyst, suggesting the coexistence of multiple active sites for cobalt-iron oxides, as experimentally reported.^32^

We note that, apart from stoichiometric coverage shown in Figures 2 and 3, we also tried off-stoichiometric patterns, with excess H on surface oxygens or excess O bridge atoms for both catalysts (these additional configurations are illustrated in Figures S3 and S4 of the Supporting Information) but observed that off-stoichiometric patterns were not favored under realistic/reaction conditions (*U* = 1.56–1.63 V), as can be seen in the energetics of Figures S3 and S4 (see the Supporting Information for details).

### 3.2 OER Mechanisms

#### 3.2.1 NiFe_2_O_4_

**Figure 4:**
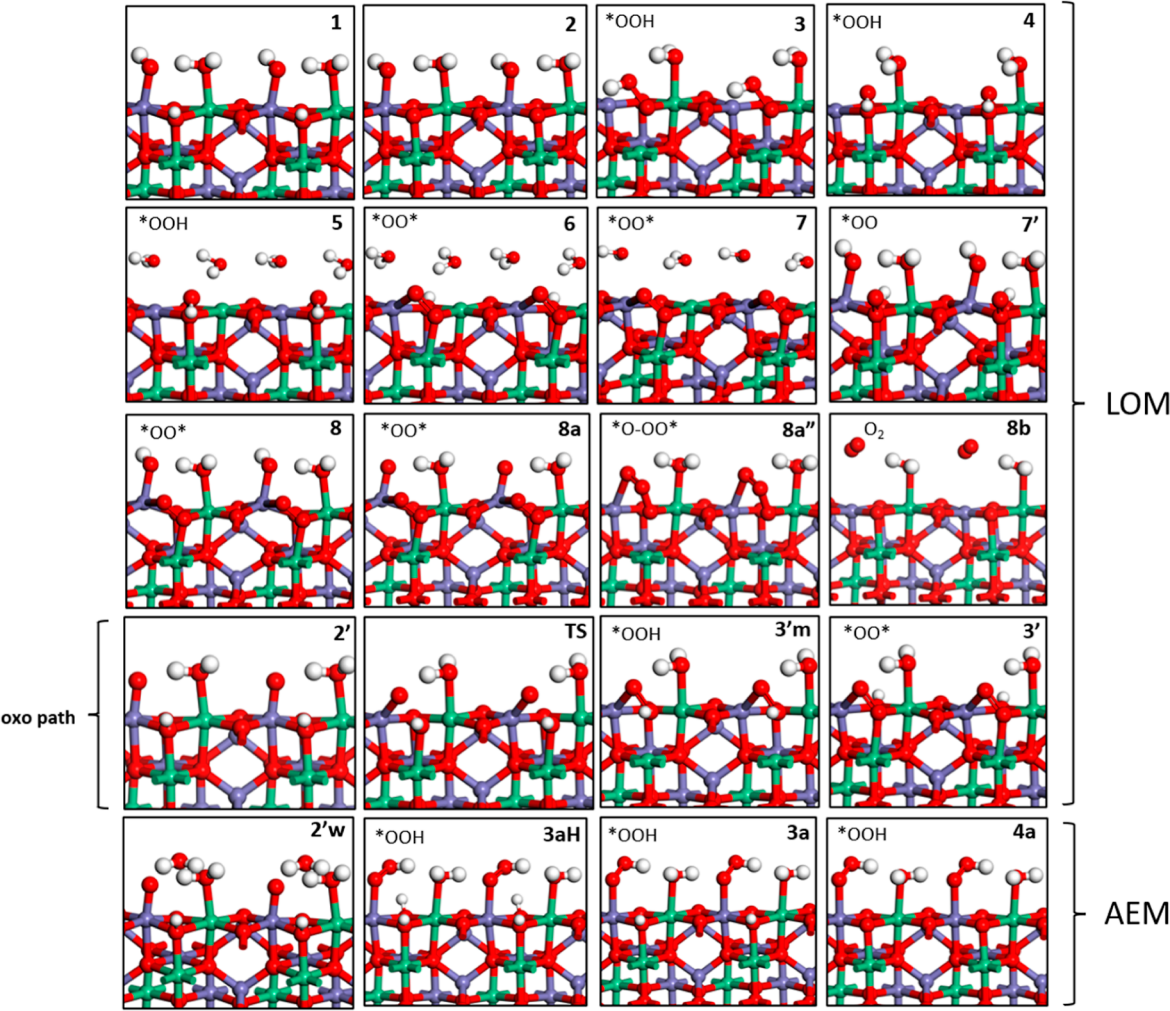
Optimized structures of the OER intermediates on (001) NiFe_2_O_4_. Oxygen, hydrogen, iron, and nickel atoms are colored red, white, violet, and green, respectively. Alternative notations on top/left corner of each states indicating the coverage on Fe, Ni, O1, and O2 sites are given in the Supporting Information, Figure S14.

**Figure 5:**
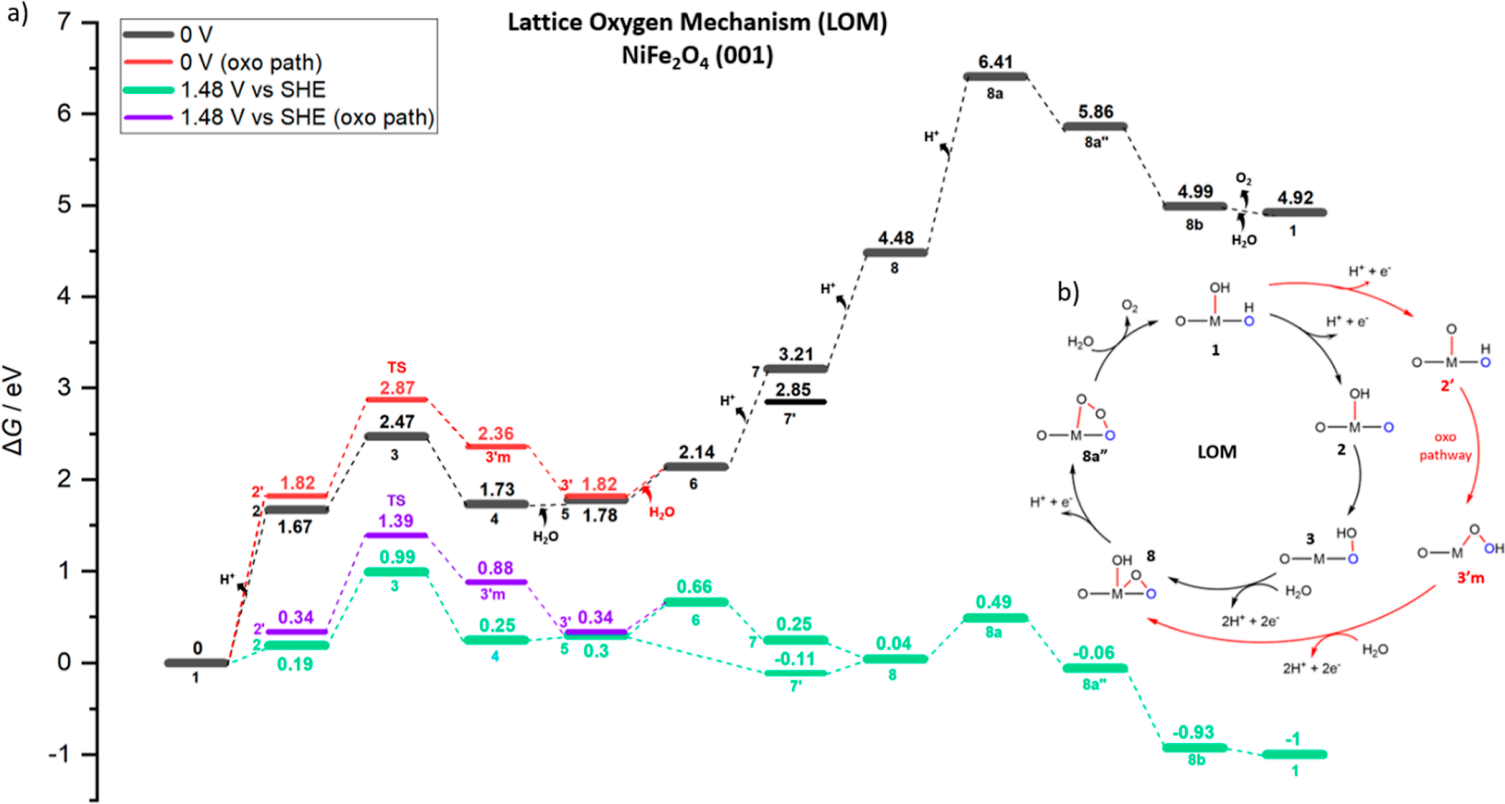
(a) Free Energy (*G*, eV) profiles that represent the LOM catalytic cycle of the OER on (001) NiFe_2_O_4_ at *U* = 0 V (black, red for the oxo path) and *U* = 1.48 V (green, purple for the oxo path) *vs* SHE. (b) Overarching mechanistic catalytic cycle labeled with the same notations on the intermediates used in the free energy profile and Figure 4.

In the following, we describe the calculated pathways for OER on the NiFe_2_O_4_ (001) surface, with the optimized structures illustrated in Figure 4 and the free-energy profile shown in Figure 5. Our description partially overlaps that proposed in ref (27), with the difference that in addition to four intermediate system in previous work, we elucidate an OER with two possible adopted mechanisms (LOM and AEM) and investigate both O–O bond formation and O_2_ liberation steps. Briefly, in the LOM, O–O formation takes place through direct coupling between an oxygen adsorbed on a metal site and a lattice oxygen, whereas in the AEM, O–O formation takes place on oxygen adsorbed on metal sites only. Illustrated pictorial OER mechanisms for acidic and basic schemes are given in the Supporting Information, Figure S5.

We start the OER catalytic cycle from the lowest-energy-dissociated water coverage state (Figure 2b) as the first intermediate, also named state **1** from now on (see Figure 4 for a pictorial illustration). First, a proton is released from the lattice oxygen (O1), ending up in state **2** with a free lattice oxygen that, together with the adsorbed OH (*OH) on Fe, will initiate the HO–O bond formation in the next step. An alternative to the path in which O–O bond formation starts from state **2** is to release the first proton from *OH to form an oxo state (*O) on Fe, named state **2′**, and the oxo adsorbate can interact directly with a protonated lattice oxygen O1–H to form an O–OH bond: we call this path “oxo path,” and it turns out that this version of the LOM mechanism represents a less favored pathway (Figure 5). In the lowest-energy LOM path, the adsorbed OH (*OH) on Fe in state **2** approaches the free lattice oxygen (O1) to make an O–OH bond on lattice O1 in state **3**. In our estimates, state **3** with its *OOH species on the surface is associated with the rds barrier: we call it the TS-like structure because it corresponds to a local minimum state, not a saddle point, but with a very low intrinsic barrier, as we discuss next. To quantify the barrier to reach this state, we performed NEB calculations, including CI, for estimating saddle points. However, CI NEBs mostly failed on this complex spin system, possibly because of spin transition and/or rotation from (*OH, state **2**) to (*OOH, state **3** and **4**). Simple NEB schemes (without CI) instead converged and are reported in the Supporting Information, see Figure S6 for state **2** to state **3**. In these simulations, the NEB algorithm did not find a sizeable barrier from state **2** to state **3**, so that the barrier can be assumed to coincide with the reaction energy, that is, 0.8 eV (see Figure S6). Sampling a direct path from state **2** to state **4**, the NEB algorithm found a steep saddle point located at 0.95 eV, which is energetically and geometrically similar to the local minimum state **3** (TS-like structure), see Figure S7. We thus use the TS-like structure, state **3**, to estimate the barrier for this OER mechanistic pathway. As for the oxo path, CI-NEB for O–O formation from state **2′** to **3′m** converged (Figure S8) and is probably numerically more stable because spin transition is not involved as much as in state **3** (*OOH). CI-NEB predict the **TS** as 1.05 eV higher than state **2′** (note that this barrier is 0.63 eV in a NEB without CI scheme, Figure S8). After state **3′m**, the proton on lattice O1 shifted to lattice O2 in state **3′**, and then oxo pathway connects to state **6** after water absorption on the Fe site left vacant.

In the lowest-energy LOM pathway, the reason why state **4** is significantly more stable than state **3** is that the *OO-H tail makes a hydrogen bond with the lattice oxygen (O2) in the neighboring cell along the y direction (see Figure S2 for a side image of **4**), and the position of the −OH resembles that of a tetrahedrally coordinated-Fe in the bulk. Continuing the OER reaction path, in state **5** a water molecule comes in to adsorb onto the undercoordinated Fe cation, after which the hydrogen of the (*OO–H) species is shifted to a lattice oxygen of neighboring cell in the y axis to protonate the surface, leaving an (*O–O*) species between surface metals in state **6**. Then, after a second deprotonation of the back lattice oxygen in state **6**, state **7** is formed, and the adsorbed water molecule on Fe releases the third proton into the solution phase, transforming into an adsorbed OH in state **8**. In the alternative route, state **5** is connected directly to state **7′** by losing a proton from the water adsorbed on the Fe site, and H of *OOH shifts to lattice O2 by leaving an uncoordinated *OO on lattice, and then losing the proton on lattice O2 and *O–O* coordination results in state **8**. After the release of a fourth proton from *OH on Fe (state **8a**), *O bends down to make a second bond with the surface (*O–O*) in state **8a″**, then finally, O_2_ is liberated from the surface, as shown in **8b**, followed by dissociative water adsorption to re-form state **1**. Note that there is no barrier during the O_2_ formation steps from 8a to 8b (see the corresponding NEB profile in Figure S9).

For the AEM pathway (last row in Figure 4), continuing with the oxo structure state **2′**, explicit water approaches the oxo site on Fe and simultaneously yields a proton on the lattice oxygen O2, forming *OOH on Fe as a new O–O bond in state **3aH**. Then, surface deprotonations occur one by one in states **3a** and **4a**. After a fourth and final deprotonation from *OOH to *OO on Fe in **4a**, O_2_ is released to reach state **8b**, where AEM reconnects to the LOM path, then followed by dissociative water adsorption to re-form state **1**.

In Figure 5, the so-derived free energy profile for OER on NiFe_2_O_4_ is shown. In black in Figure 5, we report the free energy profile under standard conditions (pH = 0, *T* = 298.15 K, *P* = 1 bar, 0 V bias); in red the oxo path at 0 V, whereas in green (and purple for the oxo path) the free energy profile at an overpotential of 0.25 V (*i.e.*, *U* = 1.48 V *vs* SHE) is shown. In detail, a 1.48 *U* (*i.e.*, electrode potential) is applied to the elementary steps that involve a proton-electron transfer (*i.e.*, deprotonation steps), occurring in four steps during the OER catalytic cycle, that is, from state **1** to **2**, from state **6** to **7** (or from state **5** to **7′**), from state **7** to **8** (or state **7′** to **8**), and from state **8** to **8a**. Here, we focus on the reaction barrier that stems from the state **3**, where O–O bond formation occurs (*i.e.*, the TS-like structure). The significant barrier while forming this key bond is 0.80 eV, according to our estimate *via* the TS-like structure. The barrier for the oxo pathway requires 1.05 eV as from state **2′**, this step (**2′** to **TS**) is also bias-independent, and it thus seems unfavorable. To the best of our knowledge, there are no predictions in theoretical studies of the critical barrier steps that determine the OER reaction rate on NiFe_2_O_4_ spinel structures, so we do not have data to compare with. As for O_2_ liberation process, the fourth and final proton release from state **8** to **8a** requires a costly step at 0 V bias by 1.93 eV by reaching a terminal oxygen (*O) generation on Fe. However, this deprotonation step is facilitated by a high electrochemical potential, requiring a barrier of 0.45 eV at 1.48 V bias, as shown in the green energy profile in Figure 5. Then, the system is stabilized by 0.55 eV as (*O) connects to surface oxygens in state **8a″** and continues to be further stabilized, leaving the triple-connected structure (by 0.44 eV), then finally closing the cycle.

**Figure 6:**
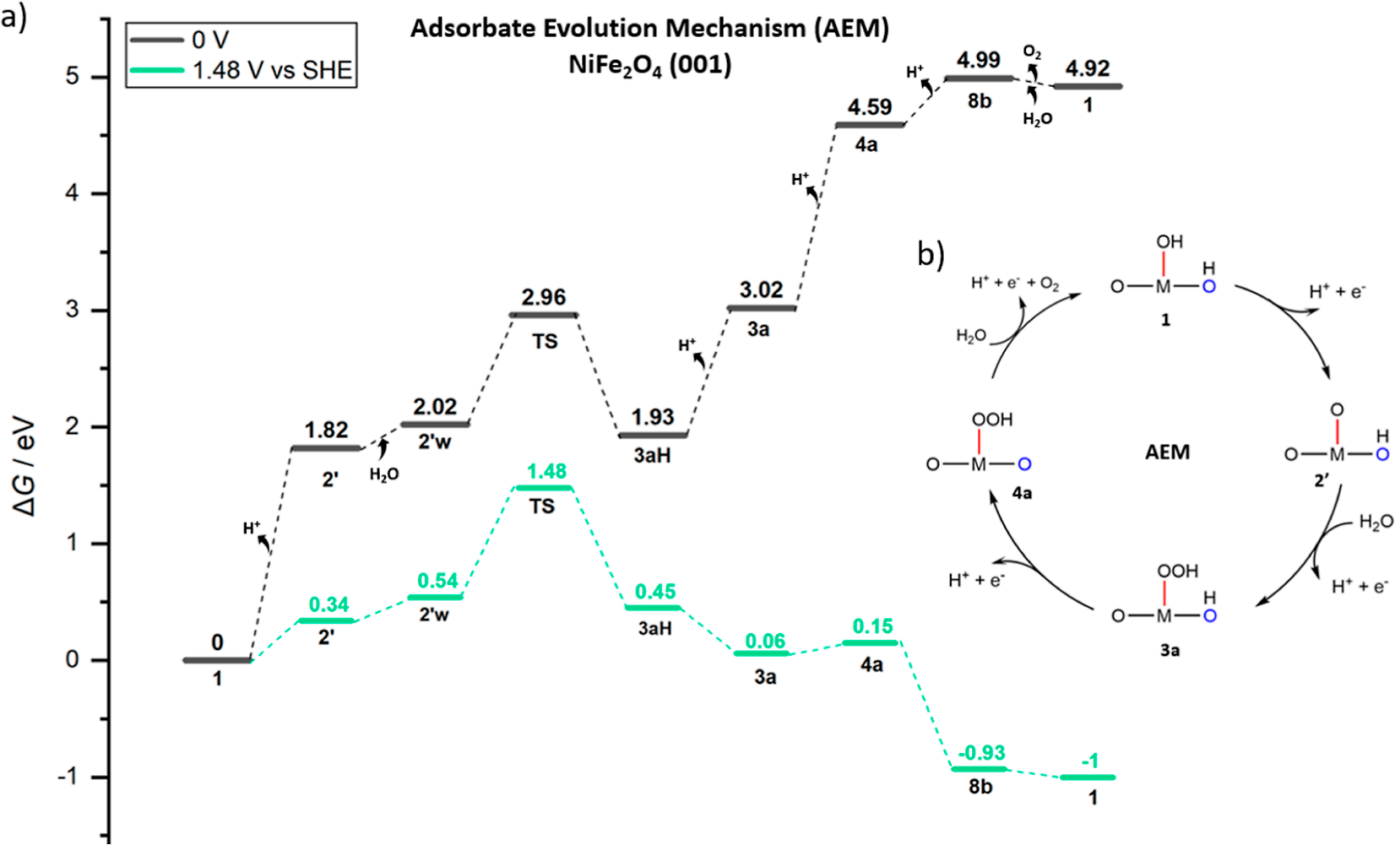
(a) Free energy (*G*, eV) profiles that represent the AEM catalytic cycle of the OER on (001) NiFe_2_O_4_ at *U* = 0 V (black) and, *U* = 1.48 V (green) *vs* SHE. (b) Overarching mechanistic catalytic cycle labeled with the same notations on the intermediates used in the free energy profile and Figure 4.

In Figure 6, free energy profile for the AEM pathway is illustrated. In addition to 0 V (black), the free-energy profile at *U* = 1.48 V versus SHE is also shown in the figure. A higher barrier of 0.94 eV from **2′w** to **TS** (see TS on Figure S10) as a bias-independent step (with the TS at 1.48 eV at 1.48 V) compared to the LOM mechanism is predicted.

Overall, therefore, the LOM pathway results to be a lower-barrier pathway compared to the AEM on this catalyst, in agreement with what was suggested in the previous literature on spinel oxides.^64−66^

With the reaction barriers so derived as a function of the electrochemical potential, we can obtain reaction rates and currents using the classical transition state theory. To estimate a computationally derived TOF of catalytic cycles, transition state theory (TST) is commonly used
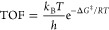
where *k*_B_ is the Boltzmann constant, h is the Planck constant, R is the universal gas constant, and T is the temperature in K, while —Δ*G*^⧧^ is the overall total barrier corresponding to the difference in free energy between the resting state and highest saddle point along the reaction path (*i.e.*, of the rds). In the lowest-barrier LOM pathway in Figure 5, the barrier responsible for O–O bond forming (**2** to **3**) is 0.80 eV and for the oxo path (**2′** to **TS**) is 1.05 eV, and these steps are bias-independent, while the first (**1** to **2**, for oxo: **1** to **2′**) and last (**8** to **8a**) electrochemical steps require 1.67, 1.82, and 1.93 eV, respectively, at 0 V (no bias), and these steps are bias-dependent, thus corresponding to a free-energy difference of 0.19, 0.34, and 0.44 eV, respectively, at 1.48.V versus SHE. The lowest-energy path then goes from state **1** to state **4***via* state **3** (nonoxo path), and the TS-like state corresponds to the rate-determining step, with an overall free-energy reaction barrier of 0.19 + 0.80 = 0.99 eV. The predicted TOF at 298 K for the path going from state **1** (*i.e.*, the resting state) to state **3** (0.99 eV) at η = 0.25 V overpotential (*U* = 1.48.V *vs* SHE) is 7.61 × 10^–4^ s^–1^. In contrast, in the energy-demanding AEM pathway in Figure 6, the overall barrier from **1** (resting state) to **TS** is 1.48 eV at η = 0.25 V overpotential (*U* = 1.48.V *vs* SHE), and the calculated TOF at 298 K is 4.08 × 10^–12^ s^–1^. The predicted TOF of 7.61 × 10^–4^ for the LOM path is rather low. However, an increase of the bias by 0.15 eV (*i.e.*, at *U* = 1.63 V *vs* SHE, η = 0.40 V) would decrease the overall barrier down to 0.84 eV from state **1** to state **3**, and thus increase the predicted TOF to 0.26 s^–1^. This prediction is not far from and compare favorably with the experimentally reported TOF’s in the study by Lim *et al.* on mesoporous NiFe_2_O_4_ spinel nanoparticles with abundant oxygen vacancies. They report that, at an overpotential of 0.40 V, the TOF of hydrogen-treated NiFe_2_O_4_ is 0.086 s^–1^, whereas the TOF of pristine NiFe_2_O_4_ and air-treated NiFe_2_O_4_ spinel nanoparticles are 0.017 and 0.053 s^–1^, respectively.^67^ Note that we predict a TOF of 0.081 s^–1^ at an overpotential of 0.37 V. We remark that experimental studies report a wide variety of overpotentials for the OER depending on the synthesis method, substrates, and catalyst loading.^9^ For example, in the study by Lim *et al.*,^67^ the overpotential at 10 mA/cm^2^ of hydrogen-treated, air-treated, and pristine NiFe_2_O_4_ spinel nanoparticles were reported as 389, 410, and 496 mV, respectively. Overpotentials thus vary, with a minimum value reported by Chen *et al.* for phosphate-ion-modified (P–NiFe_2_O_4_) nanosheets, that is, 231 mV at 10 mA/cm^2^.^68^

#### 3.2.2 CoFe_2_O_4_

**Figure 7:**
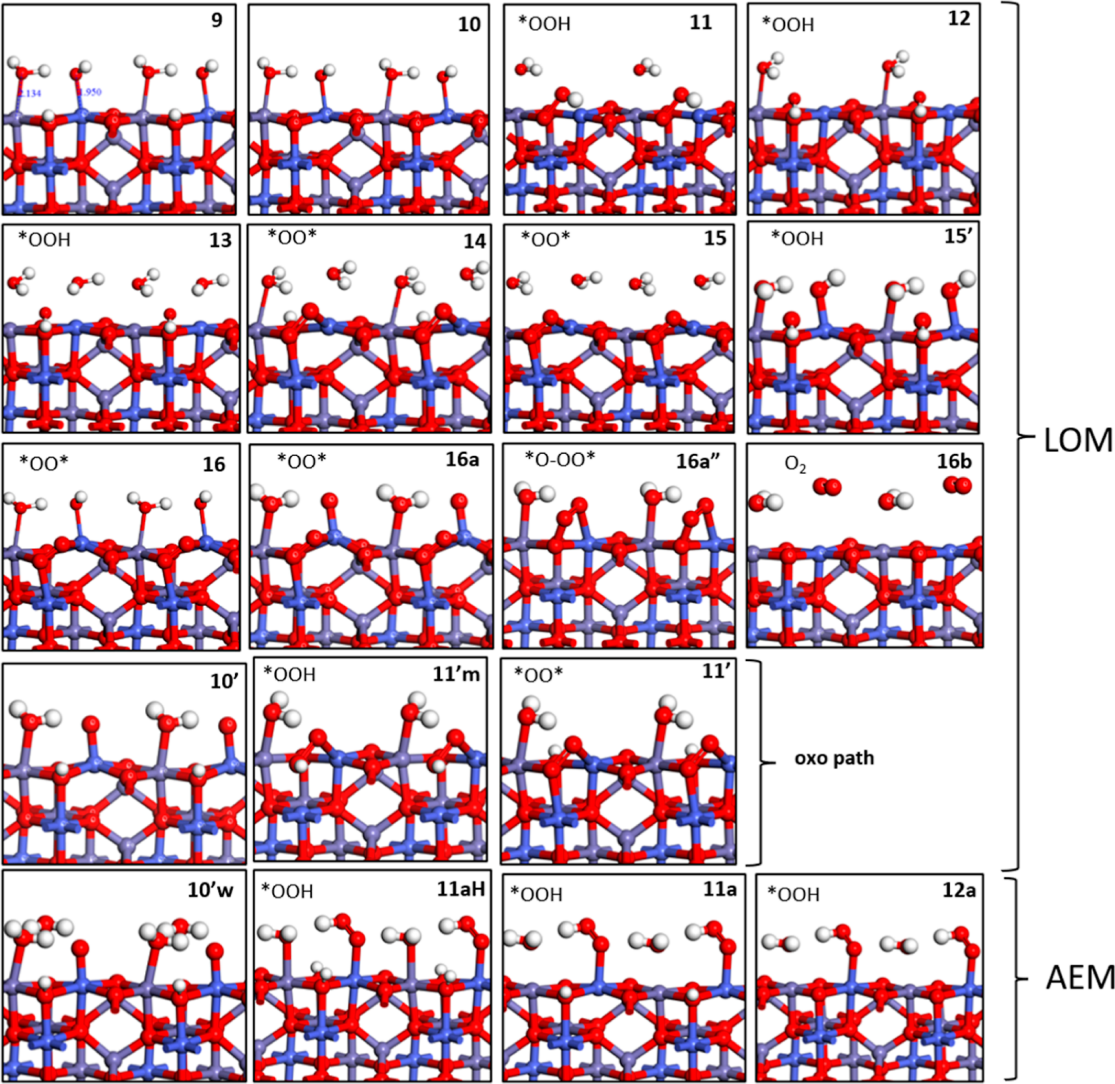
Optimized structures of the OER intermediates on (001) CoFe_2_O_4_ showing OER assisted by Co sites. Oxygen, hydrogen, iron, and cobalt atoms are colored red, white, violet, and indigo blue, respectively. Alternative notations on the top/left corner of each states indicating the coverage on Fe, Co, O1, and O2 sites are given in the Supporting Information, Figure S15.

**Figure 8:**
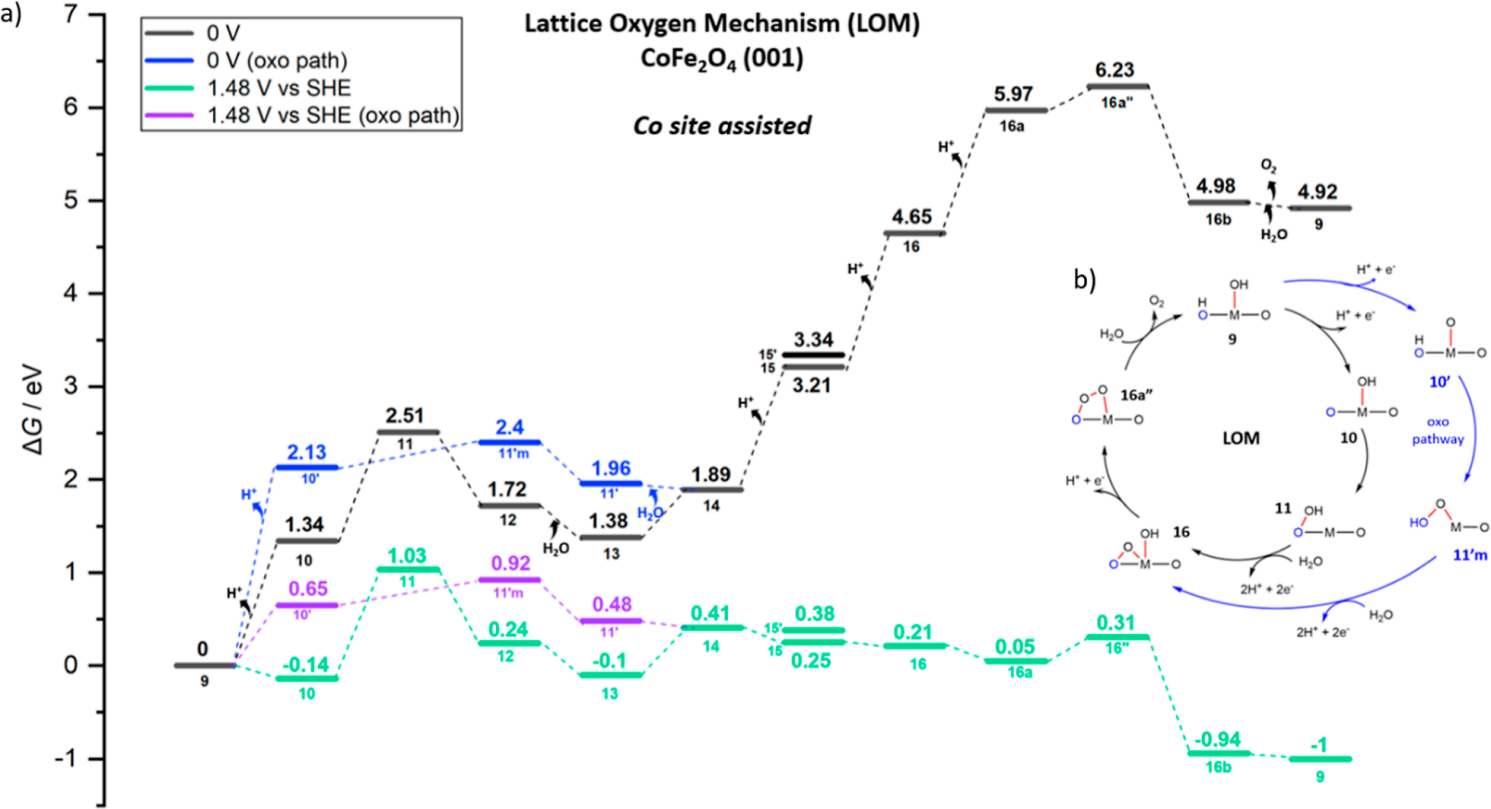
(a) Free energy (*G*, eV) profiles that represent for the LOM assisted by Co sites, OER intermediates (shown in Figure 7) of catalytic cycle on (001) **CoFe**_**2**_**O**_**4**_ at *U* = 0 V (black, blue for the oxo path) and *U* = 1.48 V *vs* SHE (green and purple for the oxo path). (b) Overarching mechanistic catalytic cycle labeled with the same notations on the intermediates used in the free energy profile and Figure 7.

**Figure 9:**
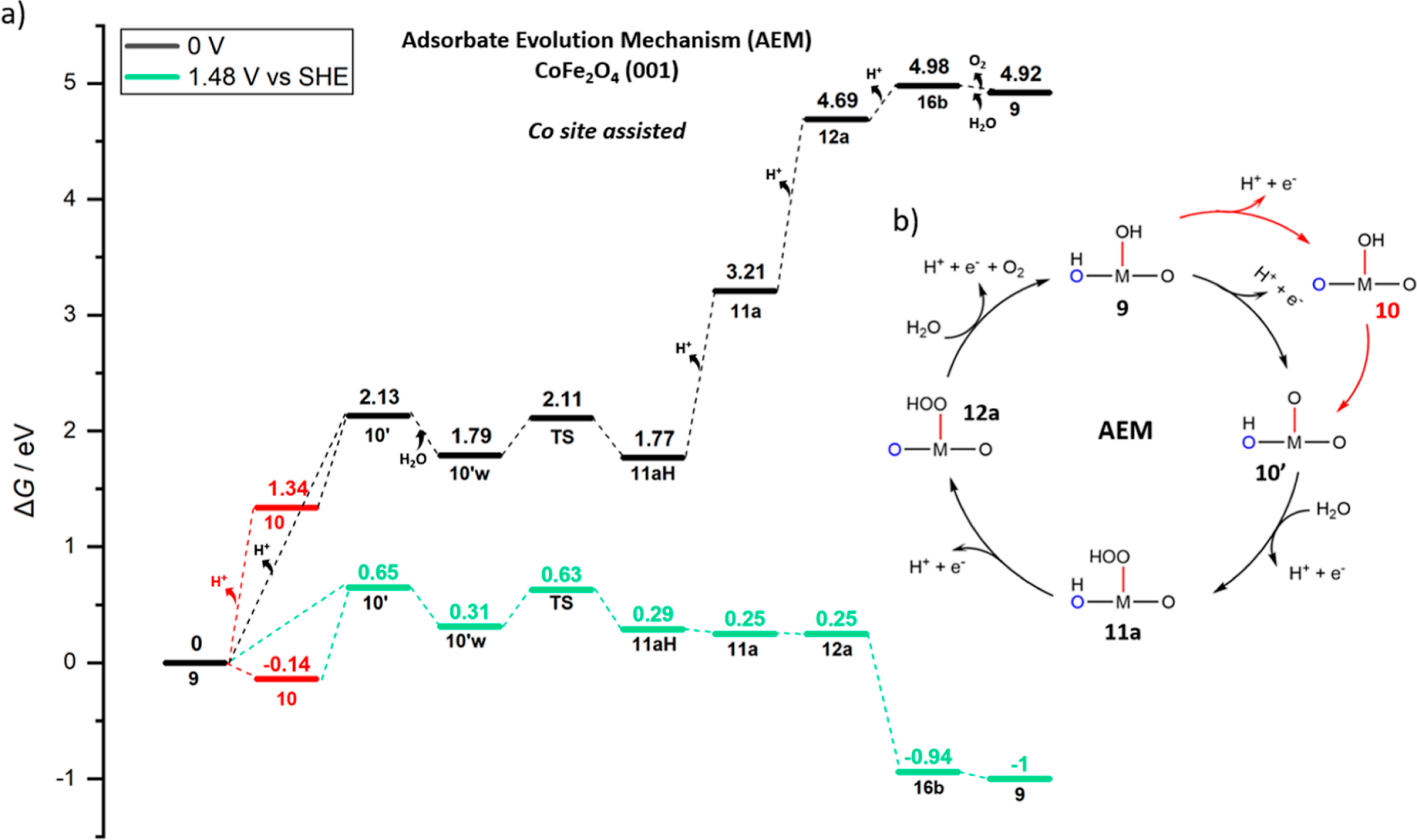
(a) Free energy (*G*, eV) profiles that represent AEM assisted by Co sites catalytic cycle of OER on (001) CoFe_2_O_4_ at *U* = 0 V (black) and *U* = 1.48 V (green). (b) overarching mechanistic catalytic cycle labeled with the same notations on the intermediates used in the free energy profile and Figure 7.

**Figure 10:**
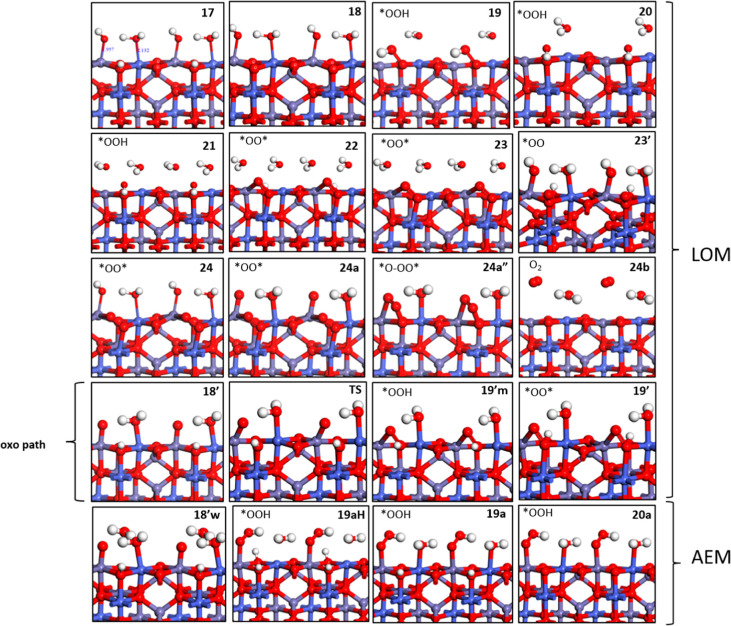
Optimized structures of the OER intermediates on (001) CoFe_2_O_4_ showing an Fe-site-assisted OER. Oxygen, hydrogen, iron, and cobalt atoms are colored red, white, violet, and indigo blue, respectively. Alternative notations on top/left corner of each states indicating the coverage on Fe, Co, O1, and O2 sites are given in the Supporting Information, Figure S16.

**Figure 11:**
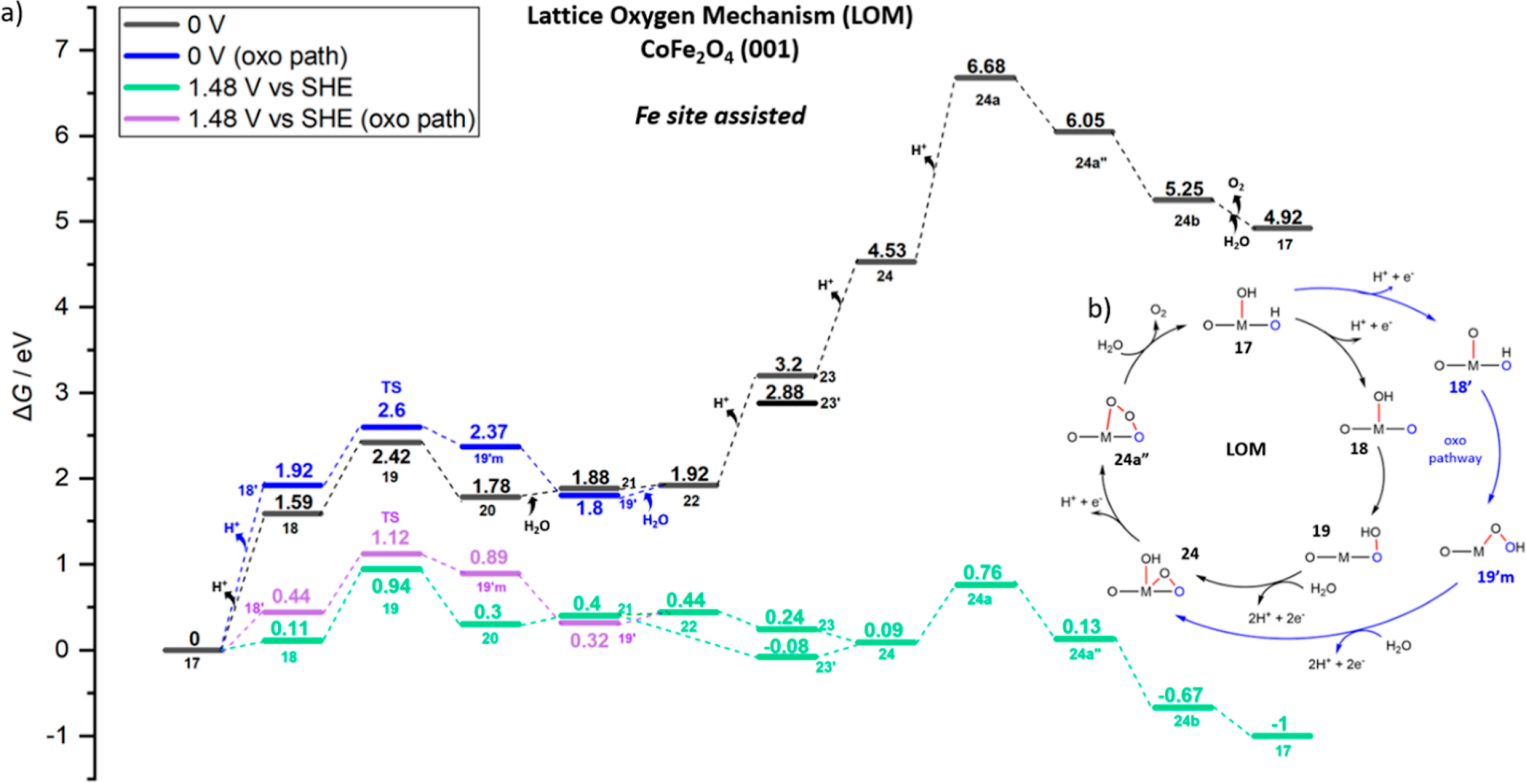
(a) Free energy (*G*, eV) profiles that represent for the LOM assisted by Fe sites, OER intermediates (shown in Figure 10) of the catalytic cycle on (001) **CoFe**_**2**_**O**_**4**_ at *U* = 0 V (black, blue for oxo path) and *U* = 1.48 V *vs* SHE (green and purple for the oxo path). (b) Overarching mechanistic catalytic cycle labeled with the same notations on the intermediates used in the free energy profile and Figure 10.

**Figure 12:**
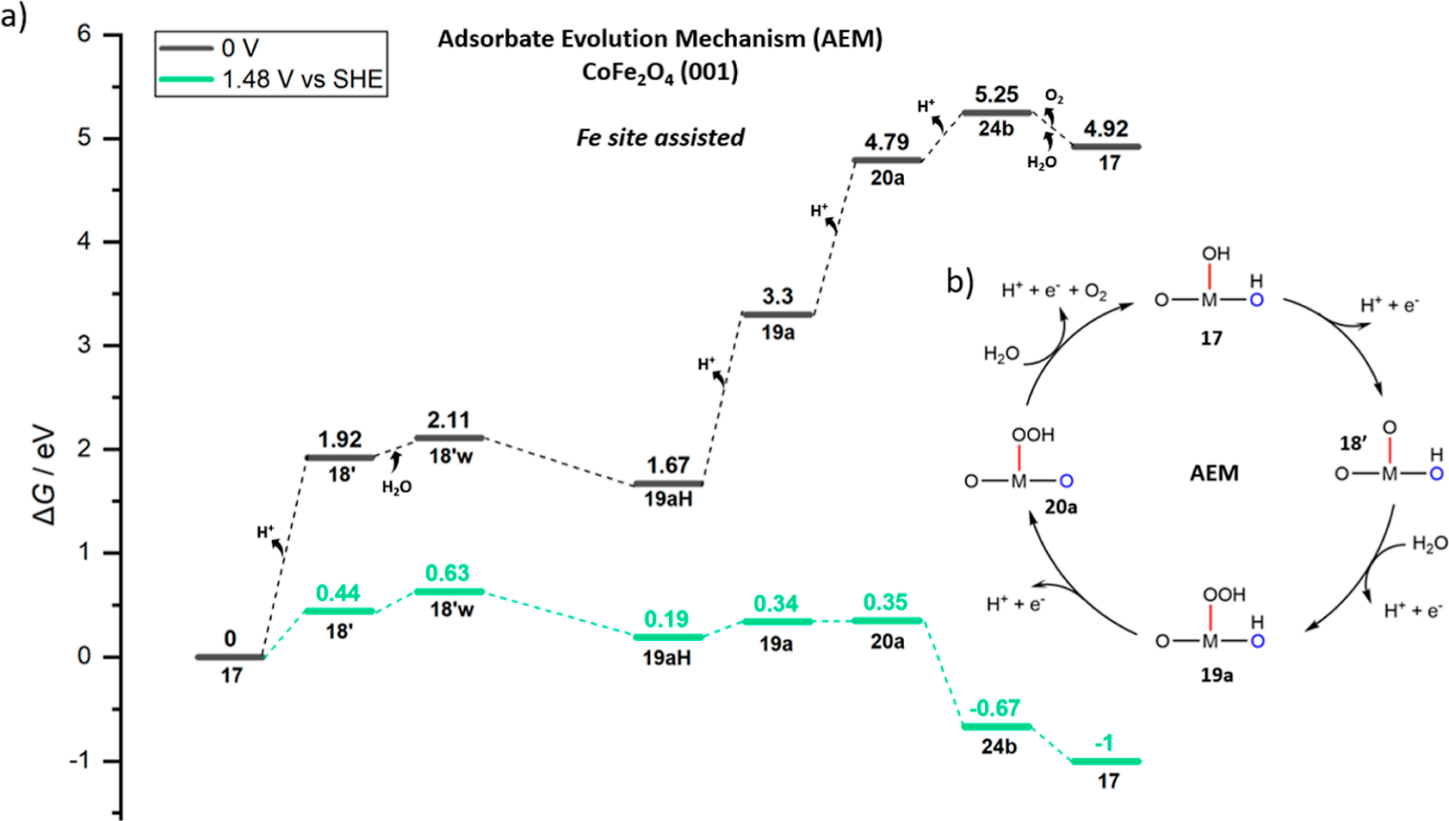
(a) Free energy (*G*, eV) profiles that represent the AEM assisted by Fe sites on (001) CoFe_2_O_4_ at U = 0 V (black) and *U* = 1.48 V (green). (b) Overarching mechanistic catalytic cycle labeled with the same notations on the intermediates used in the free energy profile and Figure 10.

To determine the reaction pathway for the OER on the CoFe_2_O_4_ (001) system, we refer to the coverage pattern shown in Figure 3 for this system. According to that, we have potentially two initial states [Figure 3b1,b2] that have similar (lowest) energies. In Figure 3b1, Fe sites exhibit H_2_O and Co sites exhibit *OH as surface-layer adsorbates, whereas in Figure 3b2, Co sites have *H_2_O and Fe sites have *OH instead. The first hydrogen release step (*i.e.*, deprotonation) allows us to explore two different reaction pathways for this catalyst, as deprotonated states result in different O–O bond formation energetics. In Figures 7 and 10, we show optimized intermediate structures considering both LOM and AEM pathways on Co and on Fe sites, respectively, while Figures 8 and 11 represent LOM free-energy profiles, and Figures 9 and 12 show AEM free-energy profiles on Co and on Fe sites, respectively, on (001) CoFe_2_O_4_.

To illustrate the OER pathways on the CoFe_2_O_4_ surface, we start with one of the lowest energetic dissociated water coverage states (Figure 3b1) as the first intermediate, which will be named as state **9** (See Figure 7) from now on, in which Fe sites are adsorbed with H_2_O and Co sites adsorbed with *OH. In state **10**, a first deprotonation takes place from the O1 lattice oxygen, and, importantly, it ends up with a much more stable deprotonated state compared to NiFe_2_O_4_. Alternatively, in the oxo path of the LOM pathway on this catalyst, the first deprotonation occurs on *OH adsorbed on Co and results in an oxo *O on the cobalt site, however, corresponding to a high-energy (2.13 eV) state **10′**, making it unfavorable. From state **10**, O–O bond formation occur as *OO-H on the surface with participating *OH on Co site to state **11**, a local minimum that we interpret again as the *TS-like structure,* as discussed above for state **3**. After the O–O bond is formed, *OO-H reorients itself in state **12** and gets stabilized by making a hydrogen bond with a lattice oxygen in neighboring cell along the y direction as for state **4** above. In state **13**, a water molecule comes to adsorb on an octahedrally coordinated surface Fe site and stabilizes the energy by 0.34 eV (Figure 8) in contrast to the NiFe case in which we found a small destabilization from state **4** to state **5** by 0.05 eV. When the hydrogen of *OO-H shifts to the lattice oxygen in the neighboring unit cell, a (*O–O*) species is formed between lattice surface atoms in state **14**. Then, consecutive deprotonation steps occur, connecting the states **14** to **15** from the surface and **15** to **16** to make *OH on Co. Finally, to prepare the O_2_ liberation step, a fourth and last proton is lost from the adsorbed *OH on Co to create a terminal oxygen (*O) in state **16a′**, followed by leaning down to connect with surface-bounded oxygens in state **16a″** that leads to O_2_ liberation from the surface in state **16b** by breaking the previous triple coordination with the surface. In order to close the cycle from **16b** to **9**, this step is followed by dissociative water adsorption on the exposed Co to re-form state **9**.

In Figure 8, the energetics of OER intermediates (from state **9** to **16b**) are shown in the free energy profile to illustrate this first catalytic cycle on CoFe_2_O_4_. Again, the black profile (blue for oxo path) shows the energetics at 0 V under standard conditions, whereas in the green (purple for oxo) profiles, an overpotential of 0.25 V (*i.e.*, *U* = 1.48 V *vs* SHE) is applied to predict the reaction free energies.

As the most important observation and comparison with the previous NiFe case, the first deprotonated state (state **10**) in this path is much more stable than in the NiFe_2_O_4_ pathway shown earlier, and a large barrier of 1.17 eV is required from state **10** to **11** (TS-like structure). Indeed, the significant highest point (TS-like structure, state **11**) that stems from (*OOH) formation resulted in an energy of 1.03 eV with respect to state **9**, significantly higher than in the corresponding OER path on NiFe_2_O_4_. The NEB for **10′** to **11′m** for the oxo path did not converge, but it seems unlikely to produce a transition state lower that of state **11**, considering that the final state (state **11′m**) is already close to the energy of state **11**. As we will also show below in Figure 9, the energy of state **10′** is 0.65 eV at *U* = 1.48 V versus SHE, and it already corresponds to the highest point along the AEM path, whereas the final state of the missing NEB (state **11′m**) in the LOM pathway is already 0.92 eV at *U* = 1.48 V versus SHE (Figure 8). There is also a smaller barrier (0.51 eV) observed from state **13** to **14** in which the hydrogen bond interaction between lattice oxygen (O2) breaks down with (*O–O*) formation on surface in state **14**. As for the fourth and final deprotonation step (state **16** to **16a**), it requires less energy (1.32 eV at 0 V bias, −0.16 eV at 1.48 V) to obtain terminal *O on the cobalt site (**16a″**) than in the NiFe_2_O_4_ pathway (**8** to **8a**). As the state **16a** is more stabilized, *O bending down to surface oxygens in the state **16″** requires a small barrier as 0.26 eV.

In Figure 9, the free energy profile for the AEM pathway is illustrated at *U* = 0 V (black) and *U* = 1.48 V (green). Although state **10′** requires a high energy of 2.13 eV to have oxo on Co sites, an explicit water interaction in **10′w** stabilizes the surface by 0.34 eV, and then O–O bond formation is assisted with explicit water leaving its proton to a surface oxygen (O2) to give rise to *OOH on Co sites. According to NEB calculations (not-CI), the barrier required for this O–O formation step was found to be 0.32 eV. This makes the AEM pathway more favorable than the LOM pathway, when OER is assisted by Co. After that point, surface deprotonations occur one by one in state **11a** and **12a**. After a fourth and final deprotonation from *OOH on Co in **12a**, O_2_ releases in state **16b** (AEM connects to LOM here), followed by dissociative water adsorption to re-form state **9**.

The AEM pathway gives the lowest-energy intermediates and barrier for CoFe_2_O_4_ (001) when the Co site assists the OER. The calculated TOF is based on the barrier (Δ*G*^⧧^) of the first electrochemical step: from state **9** to state **10′**, which is 0.65 eV at 1.48 V, to which the free-energy difference with respect to the resting state **10** (also illustrated in Figure 9) should be added for a total overall Δ*G*^⧧^ of 0.79 eV. Note that the state **10** should be considered as the resting state for all bias >1.34 V. In conclusion, at *U* = 1.48 V, the barrier from state **10** to **10′** is 0.79 eV, and the corresponding TOF will be 1.81 s^–1^, to be compared with the one predicted on NiFe_2_O_4_ (001) of 0.26 s^–1^ at 0.40 V overpotential. Here, we recall that Goddard *et al.* recently reported that the favorable mechanism for the OER on Co single sites is AEM for the Co doping-TiO_2_ catalyst, unlike the dominant LOM in perovskites and oxides, and at 1.53 and 1.63 V (300 and 400 mV OER overpotential), the computationally predicted TOF with the grand canonical QM (GCQM) method were reported to be 13.7 and 307.4 s^–1^, respectively, alongside experimentally reported values of 6.6 ± 1.2 and 181.4 ± 28 s^–1^.^69^

As for the energy-demanding LOM pathway for the Co-site-assisted OER in Figure 8, the overall barrier, which is bias-independent, from resting state **10** to state **11** is 1.17 eV, and the corresponding TOF is 6.97 × 10^–7^ s^–1^.

In Figure 10, we show alternative optimized structures for CoFe_2_O_4_, in which Co sites are exposed to H_2_O this time (as the Ni sites in the NiFe_2_O_4_ scheme), and Fe sites assist OER. In Figure 11, free energy profiles for LOM pathways are shown. As illustrated in Figure 3b2, this pattern of dissociated water coverage also gives the lowest energetic as in Figure 3b1 (*aka* state **9**). From now on, we call this (Figure 3b2) configuration state **17** as a starting intermediate, and again we investigate two routes after state **17**, including the oxo pathway of LOM. After a first proton release from lattice oxygen in state **17**, state **18** is formed 0.25 eV higher in energy compared to state **10**. In state **19** (TS-like structure), O–O bond formation occur (0.83 eV barrier) as *OO-H on the surface formed by *OH adsorbed on the Fe site, and subsequently, the surface is stabilized by 0.64 eV *via* rearrangement of the *OO-H position in state **20**. The TS-like structure (state **19**) estimate gives us the barrier at the point where the O–O bond is formed from state **18** to state **20**. Alternatively, in the oxo pathway (Figure 11), after state **17**, Fe forms the oxo and requires a bit more energy to form the intermediate **18′** (1.92 eV at 0 V, 0.44 eV at 1.48 V) in which *O on the Fe site approaches the protonated lattice oxygen O1. NEB calculations (no CI) found a barrier of 0.68 eV while forming O–OH on the lattice in state **19′m**. Note that this barrier is bias-independent. Another slight difference for this path compared to the Co-assisted OER scheme is that when a new water comes to adsorb on the empty metal site (Fe this case) in state **21**, the system is destabilized by 0.1 eV (Figure 11) in contrast to state **13** (Figure 8). Note that, in this stage of the reaction and state of the surface, a similar destabilizing trend was observed in the LOM pathway of NiFe_2_O_4_, in which the Fe site was similarly undercoordinated and able to accept water during the step from state **4** and **5**. In comparison, it seems that Co sites are comparatively more stabilized by completing their 6^th^ coordination valence with water. Then, in state **22**, after the hydrogen shifts from (*OO-H) to back lattice oxygen, an (*O–O*) species is formed on the surface with a barrier of only 0.04 eV. After the release of a second proton from the back lattice, oxygen transforms the surface into state **23**, a third proton releases from the H_2_O adsorbed on Fe leads to state **24**. As an alternative route, state **21** can connect to state **23′** by shifting the hydrogen of (*OOH) to the lattice oxygen O2 of the neighboring cell in the y direction and losing a third proton from the surface lattice O2 ends up in state **24**. The fourth and last proton release from *OH on Fe to get a terminal O (*O) (state **24a**) requires a costly step (2.15 eV at 0 V bias, *i.e.*, 0.67 eV at 1.48 V *vs* SHE), then *O bends down to connect with the surface (*O–O*) in state **24a″** and stabilizes the system by 0.63 eV. Finally, O_2_ is released from the surface by breaking down its triple coordination from state **24a″** to state **24b**, with a stabilization of 0.80 eV. After liberating O_2,_ dissociative water adsorption occurs to close the cycle and to re-form state **17**.

In Figure 12, in the case of the AEM pathway (last column in Figure 10 for optimized structures), continuing with oxo structure state **18′** (1.92 eV at 0 V), explicit water approaches the oxo site on Fe in state **18′w** and leaves one proton on lattice oxygen O2, forming *OOH on Fe as a new O–O bond with the AEM scheme in state **19aH** by stabilizing 0.44 eV. Note that NEB calculations for O–O bonding from **18′w** to **19aH** did not converge, but this mechanism is unlikely to have a barrier lower compared to the LOM pathway since the initial state of the missing NEB (**18′w**) is already at a higher energy in the AEM pathway (2.11 eV at 0 V, 0.63 eV at 1.48 V in Figure 12) compared to the initial state (state **18**) of the O–O bond formation in the LOM pathway (1.59 eV at 0 V, 0.11 eV at 1.48 V in Figure 11). Then, surface deprotonations occur one-by-one in state **19a** and **20a**. After a fourth and final deprotonation from *OOH on Fe in **20a**, O_2_ releases as in state **24b** and (AEM connects to the LOM here) then followed by dissociative water adsorption to re-form state **17**. The free energy profile is reported in Figure 12.

In the lowest-barrier LOM pathway in the Fe-assisted OER on CoFe_2_O_4_ (001), the barrier is calculated from the resting state to the state in which O–O bonding occurs. In Figure 11, the required barrier (Δ*G*^⧧^) responsible for O–O bond formation (**18** to **19**) is 0.83 eV and for the oxo path (**18′** to **TS**) is 0.68 eV, and these steps are bias-independent, but to these barriers, one should add the reaction energy of the first electrochemical step (**17** to **18**), that is, 0.11 eV at 1.48 V, and 0.44 eV at 1.48 V for the oxo path (**17** to **18′**), respectively (note that these steps are bias-dependent). For state **17** (*i.e.*, resting state) to **19** (barrier = 0.94 eV at η = 0.25 V, *U* = 1.48.V *vs* SHE), the predicted TOF at 298 K is 5.31 × 10^–3^ s^–1^, whereas at η = 0.40 V overpotential, the predicted barrier is Δ*G*^⧧^ = 0.79 eV, and the TOF at 298 K will be 1.81 s^–1^. For the oxo path: from state **17** (*i.e.*, resting state) to **TS** (1.12 eV) at η = 0.25 V (*U* = 1.48.V *vs* SHE), one predicts a TOF at 298 K of 4.86 × 10^–6^ s^–1^, while at η = 0.40 V (Δ*G*^⧧^ = 0.97 eV), TOF at 298 K will be 1.6 × 10^–3^ s^–1^. As for the AEM pathway for the Fe-assisted OER on CoFe_2_O_4_, quantitative data for TOF are not reported since the TS was not converged.

The overall predicted barrier and TOF are reasonable in keeping with the experiment. Ferreira *et al.* reported the synthesis of cobalt ferrite (CoFe_2_O_4_) powders by a proteic sol-gel green method as OER catalysts. At an overpotential of η = 400 mV, the reported TOF was 1.9 × 10^–3^ s^–1^ and 8.8 × 10^–2^ s^–1^ for gelatin based and agar–agar based samples, respectively.^70^ At the experimental level for CoFe_2_O_4_, a range of overpotentials from 266 to 490 mV @10 mA/cm^2^ were reported (along with a variety of results on the stability of these systems) up to 689 mV@100 mA/cm^2^ with catalysts obtained *via* a precipitation synthesis method.^71,72^ Lei *et al.* reported a mesoporous CoFe_2_O_4_ thin film obtained with the liquid-phase epitaxial method, which has an overpotential of 266 mV at 10 mA/cm^2^, which claimed to be a higher electrocatalytic OER than commercial RuO_2_, suggesting that the homogeneous and continuous bimetallic oxide film increased the OER performance. In the same study, the reported TOFs (at the overpotential of 330 mV) of the CoFe_2_O_4_ thin film, CoFe_2_O_4_ powder, and RuO_2_ are 0.0698, 0.0053 and 0.0037 s^–1^, respectively, and the largest TOF of the CoFe_2_O_4_ thin film indicates its highest catalytic property for the OER compared with CoFe_2_O_4_ powder and RuO_2_.^73^ Noting that, in our calculations for state **17** to **19**, at η = 0.31 V (*U* = 1.54 V *vs* SHE, Δ*G*^⧧^ = 0.88 eV) and η = 0.33 V (*U* = 1.56 V *vs* SHE, Δ*G*^⧧^ = 0.86 eV), the predicted computational TOF will be 0.0547 and 0.12 s^–1^ for CoFe_2_O_4_, respectively.

## 4 Conclusions

Herein, we modeled OER energetic pathways considering both the LOM and AEM on the (001) facet of two selected inverse spinels: NiFe_2_O_4_ and CoFe_2_O_4_. We analyzed the mechanistic pathways on these facets and discussed the role of Co’s and Fe’s sites toward the OER. First, we searched for the resting-state coverage patterns to find the lowest-energy/most-stable state that initiate the OER reaction. Both spinels favor one degree dissociated water coverage (*OH + *H, *H_2_O) on metal sites as the resting state, but in the case of NiFe_2_O_4_, Ni sites are preferentially exposed to *H_2_O, and the Fe- assisted OER was strongly preferred, whereas on CoFe_2_O_4_, the situation is more complex, with a degeneracy of (*OH + *H) distribution on Fe and Co and two parallel, independent OER paths. On NiFe_2_O_4_, a LOM pathway gives the lowest barrier (Δ*G*^⧧^ = 0.84 eV at *U* = 1.63 V *vs* SHE) for O–O bonding compared to the oxo-pathway for the LOM and AEM mechanism. The predicted TOF at 298 K and at the ideal target η = 0.25 V overpotential (*U* = 1.48.V *vs* SHE) is 7.6 × 10^–4^ s^–1^, but it increases to 0.26 s^–1^ at η = 0.40 V. On CoFe_2_O_4_, our calculations suggest that Co and Fe sites could play a synergistic role and suggest a coexistence of multiple active sites, as demonstrated by the six competing reaction pathways that we report for this catalyst. When Co sites assist the OER, the AEM pathway is favored, and, at bias *U* > 1.34 V, the rds barrier is 0.79 eV, and the corresponding TOF at 298 K is 1.81 s^–1^. When Fe sites assist the OER, a LOM pathway is favored, as in the NiFe_2_O_4_ case, and the rds barrier is 0.79 eV at η = 0.40 V (*U* = 1.63 V *vs* SHE), leading to a TOF at 298 K of 1.81 s^–1^ at η = 0.40 V overpotential. All in all, our results show that in the pristine case, CoFe_2_O_4_ may lead to lower barriers and higher TOFs compared to NiFe_2_O_4_, especially in the Co-site-assisted OER mechanism. We highlight the importance of investigating real barriers obtained with transition-state structures, as we aim in the present study. This opens the way to a much needed closer comparison and cross-validation, whence refinement, with experimental characterization, that could lead to deeper understanding and optimized design of more efficient systems. It is our hope that the broad set of OER intermediates and reaction paths presented here will help understand the complexity of the problem and trigger experimental studies, eventually providing a rational guidance for developing more efficient OER electrocatalysts.

## Supporting Information Available

Unit cells on each surface; off stoichiometric coverage patterns; OER illustrative pictorial paths for acidic and basic (alkaline) schemes; NEB simulation details, one step charged/solvent calculations; and energetics of all elementary steps of the OER on each surface (PDF)
