# Supplementary material for: Mechanisms of the Oxygen Evolution Reaction on NiFe2O4 and CoFe2O4 Inverse-Spinel Oxides
Source: ACS Catal. 2022 Jul 13;12(15):9058–73. doi: 10.1021/acscatal.2c01534 (PMC9361295; doi:10.1021/acscatal.2c01534)
Supplement: Supplementary file 1 — cs2c01534_si_001.pdf [file cs2c01534_si_001.pdf]

Supporting Information for

Mechanisms of Oxygen Evolution Reaction (OER) on $NiFe_2O_4$ and $CoFe_2O_4$ inverse-spinel oxides

Öyküm N. Avcı[1-2], Luca Sementa[3,*] , Alessandro Fortunelli[1,*]

[1] *CNR-ICCOM, Consiglio Nazionale delle Ricerche, via G. Moruzzi 1, 56124, Pisa, Italy*

[2] *Department of Chemistry and Industrial Chemistry, DSCM, University of Pisa, Via G. Moruzzi 13, 56124, Pisa, Italy*

[3] *CNR- IPCF, Istituto per i Processi Chimico-Fisici, Via G. Moruzzi 1, 56124, Pisa, Italy*

* Corresponding authors - emails: alessandro.fortunelli@cnr.it, luca.sementa@cnr.it

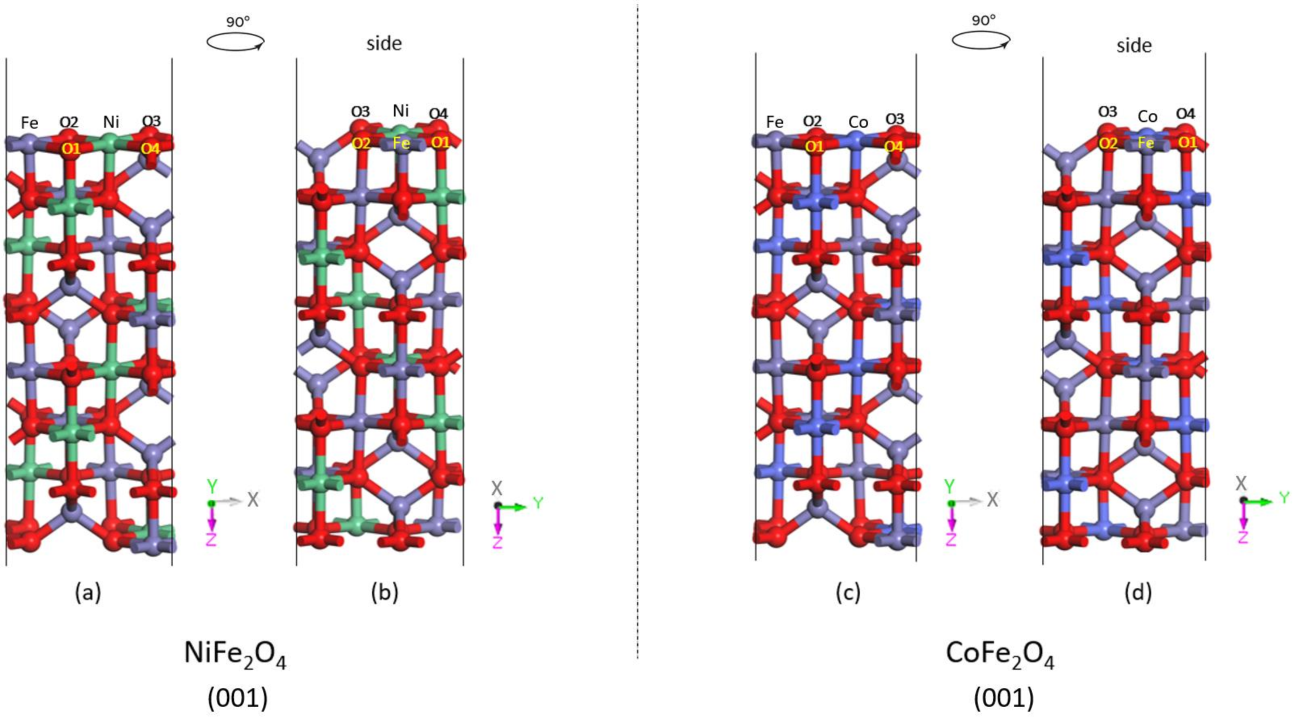

Figure S1. Unit cells of $NiFe_2O_4$ (001) and $CoFe_2O_4$ (001) surfaces in our calculations. Oxygen, iron, nickel, cobalt atoms are colored red, violet, green and indigo-blue, respectively.

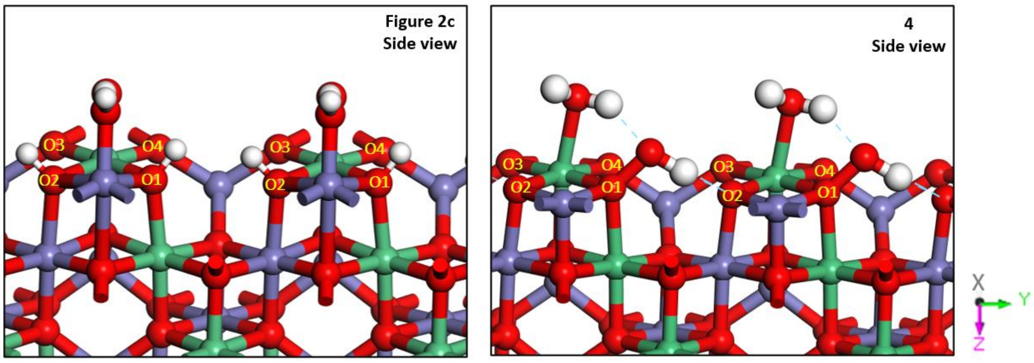

FigureS2. Side view images of Figure2c (left) and state **4** (right). Blue lines on state **4** denote the H-bonding.

1. Off-stoichiometric Configurations

As it can be seen in the energetics of **Figure S3** and **S4**, off-stoichiometric patterns hardly correspond to the resting states of the system. In **Figure S3**, in addition to the two water coverage that we considered in main text, we considered additional configurations in which surface oxygens were multiply protonated. As apparent from the energetics in **Figure S3**, these additional configurations do not correspond to the resting states of the $NiFe_2O_4$ system under realistic/reaction conditions (U =1.48-1.63 V), while two configurations are very marginally favored for the $CoFe_2O_4$ system only up to a bias of 1.56 V. Note also that configurations alternative to that in **Figure S3(f)** have been also tried (i.e. H_2O on Fe, OH on Co, and 2 surface proton) but eventually converged to the pattern of **Figure S3(f).**

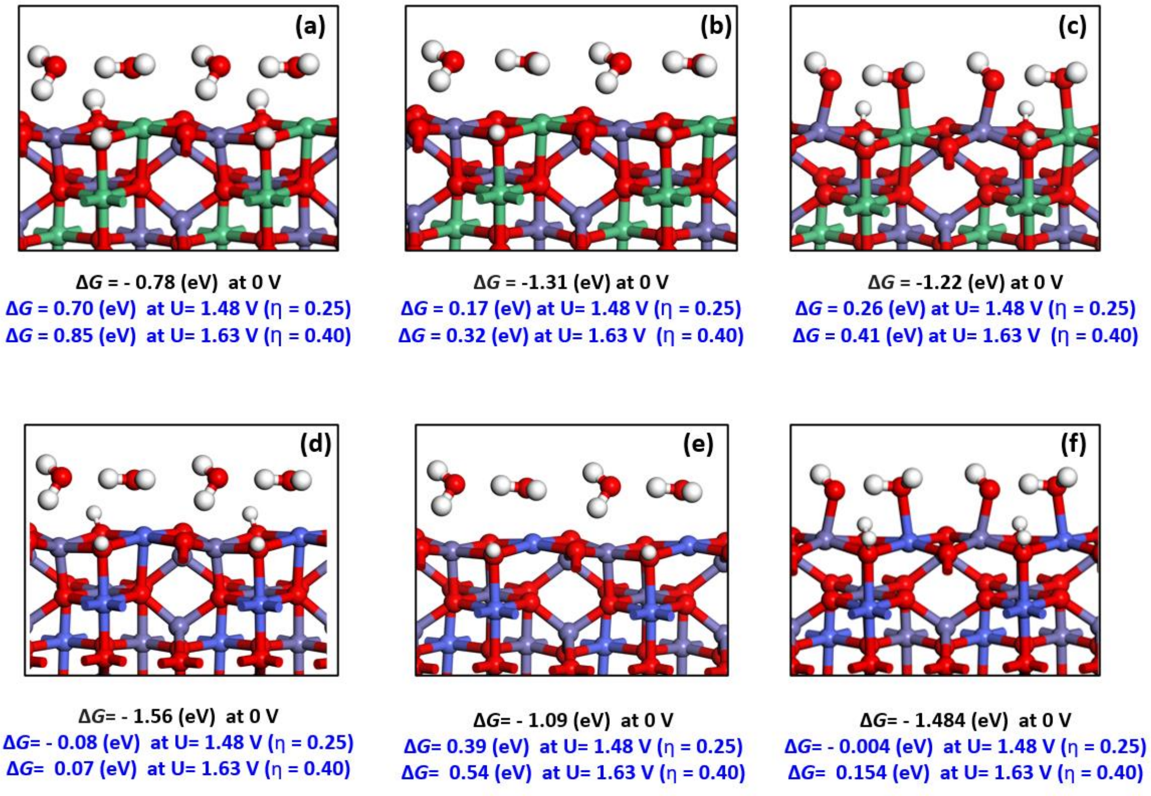

FigureS3. Off-stoichiometric coverage patterns with excess H. (a)-(c) for $NiFe_2O_4$, (d)-(e) for $CoFe_2O_4$.

As for excess O species, in **Figure S4** we report both initial and optimized geometries for both $NiFe_2O_4$ and $CoFe_2O_4$ catalysts to show that excess O's (and excess OH) are not stable during optimization, due to the fact that over-coordination on metals is not favored.

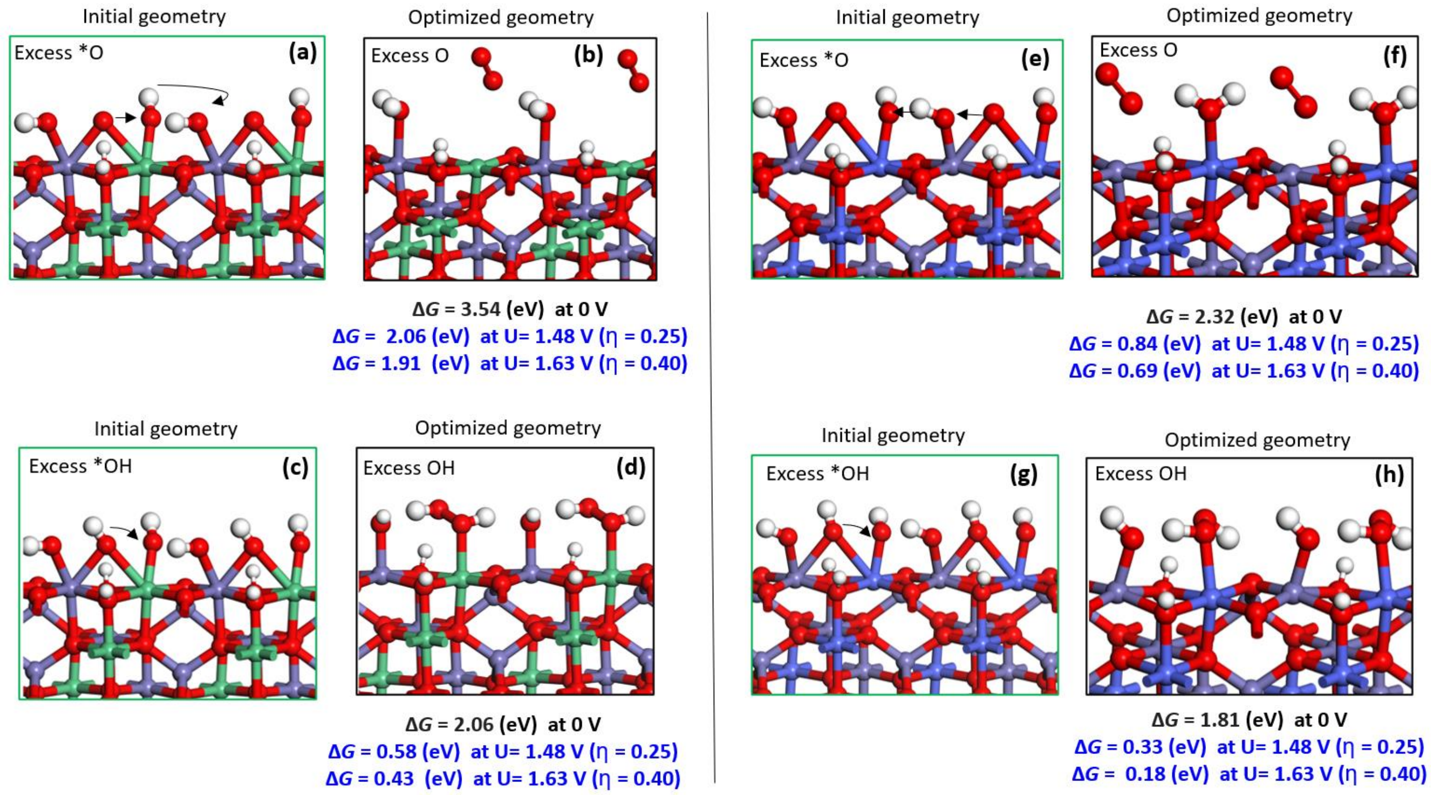

FigureS4. Off-stoichiometric coverage patterns with excess O and OH on $NiFe_2O_4$ (left) and on $CoFe_2O_4$ (right). The initial geometries for (a) excess O and (c) excess OH on $NiFe_2O_4$, black arrows show atom movements during optimizations. Optimized geometry for (b) excess O, and (d) excess OH on $NiFe_2O_4$, energies referenced to the state 1 (Figure 2b). The initial geometry for (e) excess O and (g) excess OH on $CoFe_2O_4$. Optimized geometry for (f) excess O, and (h) excess OH on $CoFe_2O_4$, energies referenced to the state 9 and 17 (Figure 3b1, b2).

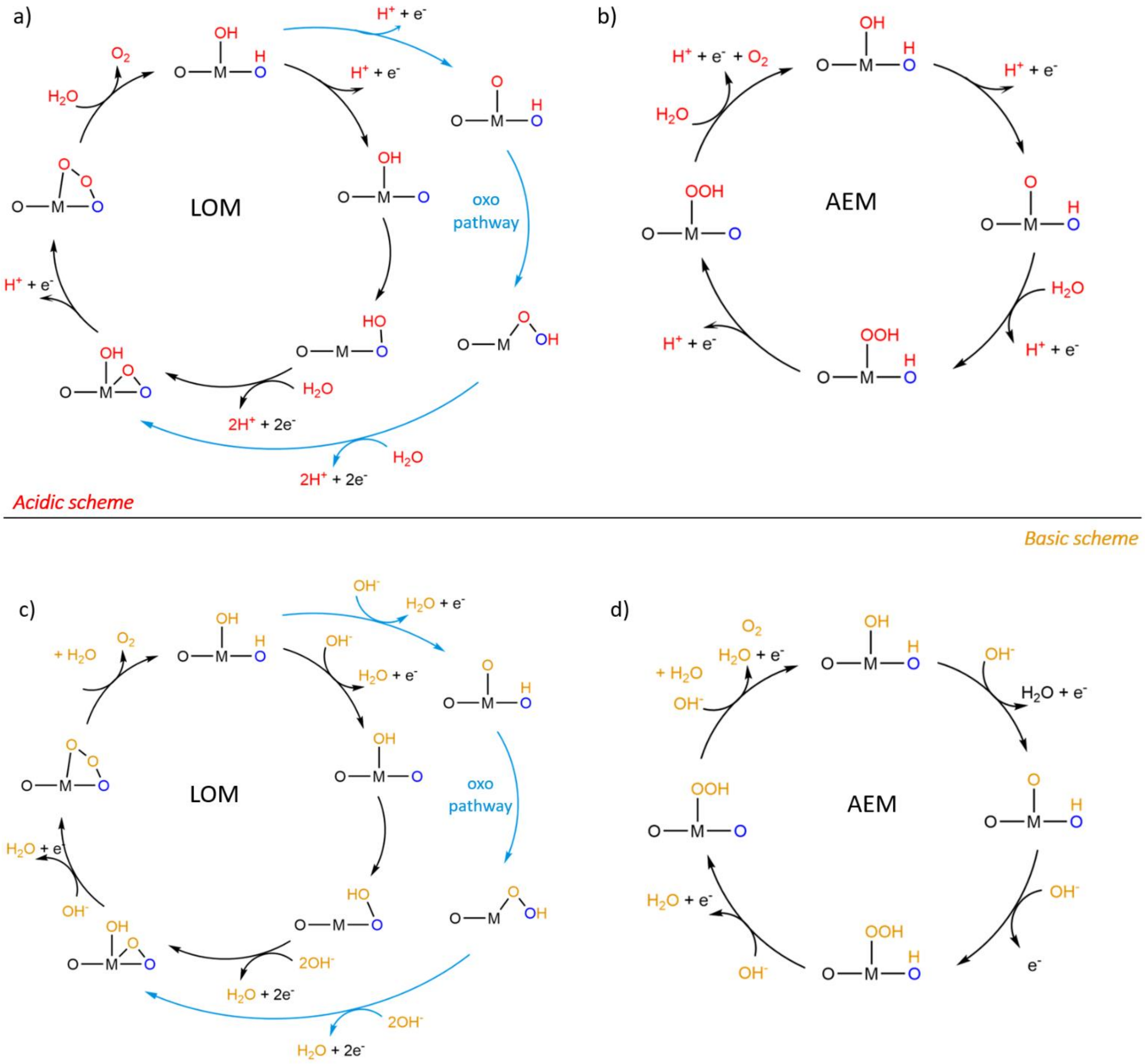

FigureS5. Illustrated OER pathways in this manuscript for acidic scheme: (a) lattice oxygen mechanism (LOM), oxo pathway of LOM shown in blue arrows ; (b) adsorbate evolution mechanism (AEM). Alternatively for basic(alkaline) scheme, (c) lattice oxygen mechanism (LOM), oxo pathway of LOM shown in blue arrows; (d) adsorbate evolution mechanism (AEM).

**$NiFe_2O_4$ (001)**						
**LOM Path**[a]						
Elemental steps	Δ*E* (eV)	Δ*H*(0→298K) (eV)	Δ*ZPE* (eV)	-*T*Δ*S* (eV)	$-\vert e\vert U$ (eV)	Δ*G* (eV)
**1** + h^+ → **2** + H^+	2.04	0.04	-0.21	-0.20	-1.48	0.19
***1** + h^+ → **2'** + H^+*	2.19	0.04	-0.21	-0.20	-1.48	0.34
**2** → **3**	0.80	0	0	0	0	0.80
2'** → **TS	1.05	0	0	0	0	1.05
**3** → **4**	-0.74	0	0	0	0	-0.74
TS** → **3'm	-0.51	0	0	0	0	-0.51
3'm** → **3'	-0.54	0	0	0	0	-0.54
**4** + H_2O → **5**	-0.69	-0.10	0.17	0.67	0	0.05
3'** + H_2O → **6	-0.42	-0.10	0.17	0.67	0	0.32
**5** → **6**	0.36	0	0	0	0	0.36
**5** + h^+ → **7'** + H^+	1.44	0.04	-0.21	-0.20	-1.48	-0.41
**6** + h^+ → **7** + H^+	1.44	0.04	-0.21	-0.20	-1.48	-0.41
**7** + h^+ → **8** + H^+	1.64	0.04	-0.21	-0.20	-1.48	-0.21
**8** + h^+ → **8a** + H^+	2.30	0.04	-0.21	-0.20	-1.48	0.45
**8a** → **8a"**	-0.55	0	0	0	0	-0.55
**8a"** → **8b**	-0.87	0	0	0	0	-0.87
**8b** + H_2O → **1** + O_2	-0.20	0	0.09	0.04	0	-0.07

[a] *oxo path in red.*

**AEM Path**						
**1** + h^+ → **2'** + H^+	2.19	0.04	-0.21	-0.20	-1.48	0.34
**2'** + H_2O → **2'w**	-0.54	-0.10	0.17	0.67	0	0.20
**2'w** → **TS**	0.94	0	0	0	0	0.94
**TS** → **3aH**	-1.03	0	0	0	0	-1.03
**3aH** + h^+ → **3a** + H^+	1.46	0.04	-0.21	-0.20	-1.48	-0.39
**3a** + h^+ → **4a** + H^+	1.94	0.04	-0.21	-0.20	-1.48	0.09
**4a** + h^+ → **8b** + H^+	0.77	0.04	-0.21	-0.20	-1.48	-1.08

$CoFe_2O_4$ (001)

**Co assisted LOM Path**[a]						
Elemental steps	Δ*E* (eV)	Δ*H*(0→298K) (eV)	Δ*ZPE* (eV)	-*T*Δ*S* (eV)	$-\vert e\vert U$ (eV)	Δ*G* (eV)
**9** + h^+ → **10** + H^+	1.71	0.04	-0.21	-0.20	-1.48	-0.14
***9**+ h^+ → **10'** + H^+*	2.50	0.04	-0.21	-0.20	-1.48	0.65
**10** → **11**	1.17	0	0	0	0	1.17
10'** → **11'm	0.27	0	0	0	0	0.27
**11** → **12**	-0.79	0	0	0	0	-0.79
11'm** → **11'	-0.44	0	0	0	0	-0.44
**12** + H_2O → **13**	-1.08	-0.10	0.17	0.67	0	-0.34
11'** + H_2O → **14	-0.81	-0.10	0.17	0.67	0	-0.07
**13** → **14**	0.51	0	0	0	0	0.51
**14** + h^+ → **15** + H^+	2.01	0.04	-0.21	-0.20	-1.48	0.16
**15** + h^+ → **16** + H^+	1.81	0.04	-0.21	-0.20	-1.48	-0.04
**16** + h^+ → **16a** + H^+	1.69	0.04	-0.21	-0.20	-1.48	-0.16
**16a** → **16a"**	0.26	0	0	0	0	0.26
**16a"** → **16b**	-1.25	0	0	0	0	-1.25
**16b** + H_2O → **9** + O_2	-0.19	0	0.09	0.04	0	-0.06

[a] *oxo path in red.*

**Co assisted AEM Path**	Δ*E* (eV)	Δ*H*(0→298K) (eV)	Δ*ZPE* (eV)	-*T*Δ*S* (eV)	-|e|*U* (eV)	Δ*G* (eV)
**9** + h^+ → **10'** + H^+	2.50	0.04	-0.21	-0.20	-1.48	0.65
**10'** + H_2O → **10'w**	-1.08	-0.10	0.17	0.67	0	-0.34
**10'w** → **TS**	0.32	0	0	0	0	0.32
**TS** → **11aH**	-0.34	0	0	0	0	-0.34
**11aH** + h^+ → **11a** + H^+	1.81	0.04	-0.21	-0.20	-1.48	-0.04
**11a** + h^+ → **12a** + H^+	1.85	0.04	-0.21	-0.20	-1.48	0
**12a** + h^+ → **16b** + H^+	0.66	0.04	-0.21	-0.20	-1.48	-1.19
**Fe assisted LOM Path**[a]						
**17** + h^+ → **18** + H^+	1.96	0.04	-0.21	-0.20	-1.48	0.11
***17** + h^+ → **18'** + H^+*	2.29	0.04	-0.21	-0.21	-1.48	0.44
**18** → **19**	0.83	0	0	0	0	0.83
18'** → **TS	0.67	0	0	0	0	0.67
**19** → **20**	-0.64	0	0	0	0	-0.64
TS** → **19'm	-0.23	0	0	0	0	-0.23
19'm** → **19'	-0.57	0	0	0	0	-0.57
**20** + H_2O → **21**	-0.64	-0.10	0.17	0.67	0	0.10
19'** + H_2O → **22	-0.60	-0.10	0.17	0.67	0	0.14
**21** → **22**	0.04	0	0	0	0	0.04
**21** + h^+ → **23'** + H^+	1.37	0.04	-0.21	-0.20	-1.48	-0.48
**23** + h^+ → **24**+ H^+	1.70	0.04	-0.21	-0.20	-1.48	-0.15
**24** + h^+ → **24a** + H^+	2.52	0.04	-0.21	-0.20	-1.48	0.67
**24a** → **24a"**	-0.63	0	0	0	0	-0.63
**24a"** → **24b**	-0.80	0	0	0	0	-0.80
**24b** + H_2O → **17** + O_2	-0.46	0	0.09	0.04	0	-0.33

[a] *oxo path in red.*

**Fe assisted AEM Path**	Δ*E* (eV)	Δ*H*(0→298K) (eV)	Δ*ZPE* (eV)	-*T*Δ*S* (eV)	-|e|*U* (eV)	Δ*G* (eV)
**17** + h^+ → **18'** + H^+	2.29	0.04	-0.21	-0.20	-1.48	0.44
**18'** + H_2O → **18'w**	-0.55	-0.10	0.17	0.67	0	0.19
**10'w** → **19aH**	-0.44	0	0	0	0	-0.44
**19aH** + h^+ → **19a** + H^+	2.00	0.04	-0.21	-0.20	-1.48	0.15
**19a** + h^+ → **20a** + H^+	1.86	0.04	-0.21	-0.20	-1.48	0.01
**20a** + h^+ → **24b** + H^+	0.83	0.04	-0.21	-0.20	-1.48	-1.02

Table S1. Energies of Elementary Steps of OER on $NiFe_2O_4$ (001) and $CoFe_2O_4$ (001) surfaces.

2. NEB Calculations

For **Figure S6 - S12**, unless otherwise stated simulations were optimized with not-CI scheme.

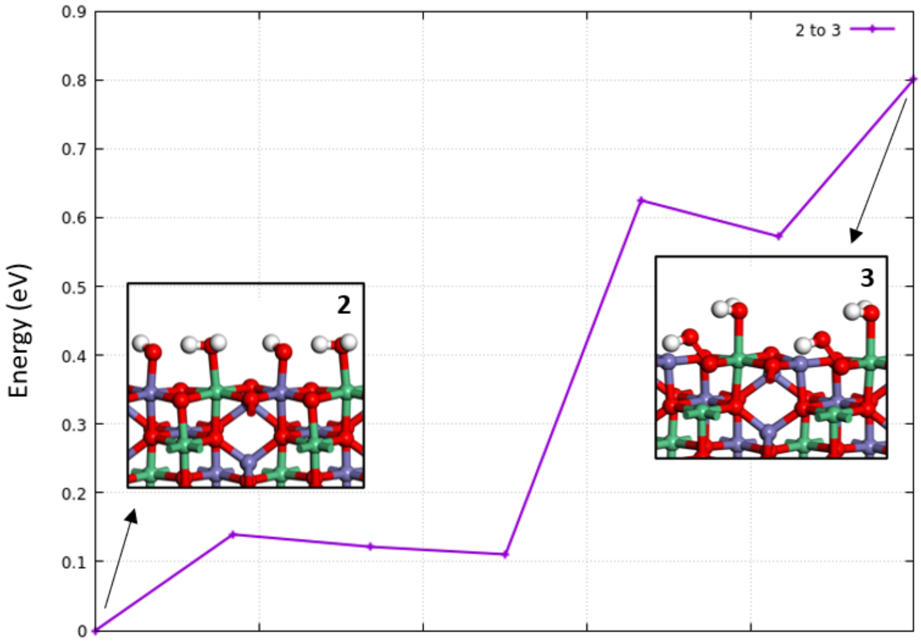

Figure S6. Nudge Elastic band (NEB) simulations for barrier search from state **2** to **3.**

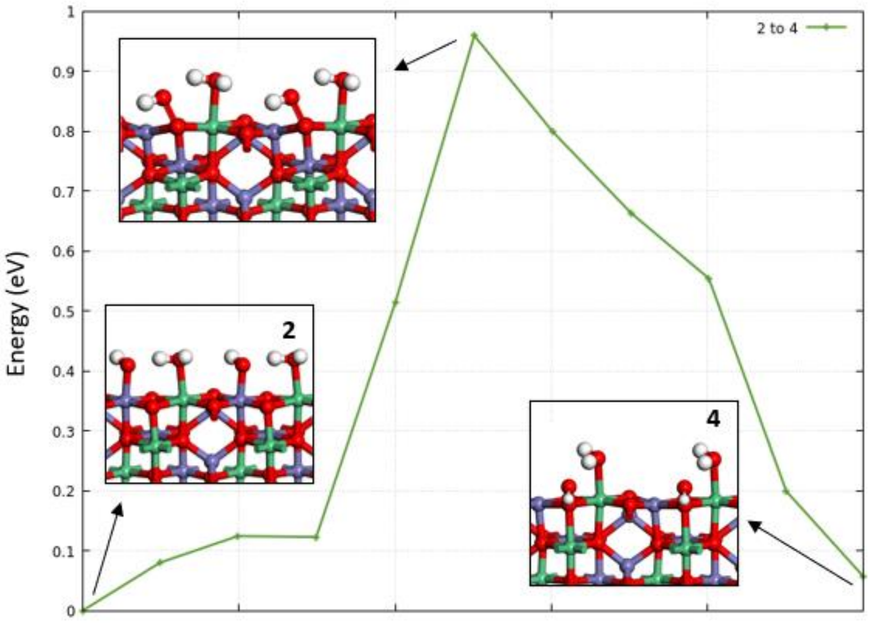

Figure S7. Nudge Elastic band (NEB) simulations for barrier search from state **2** to **4.**

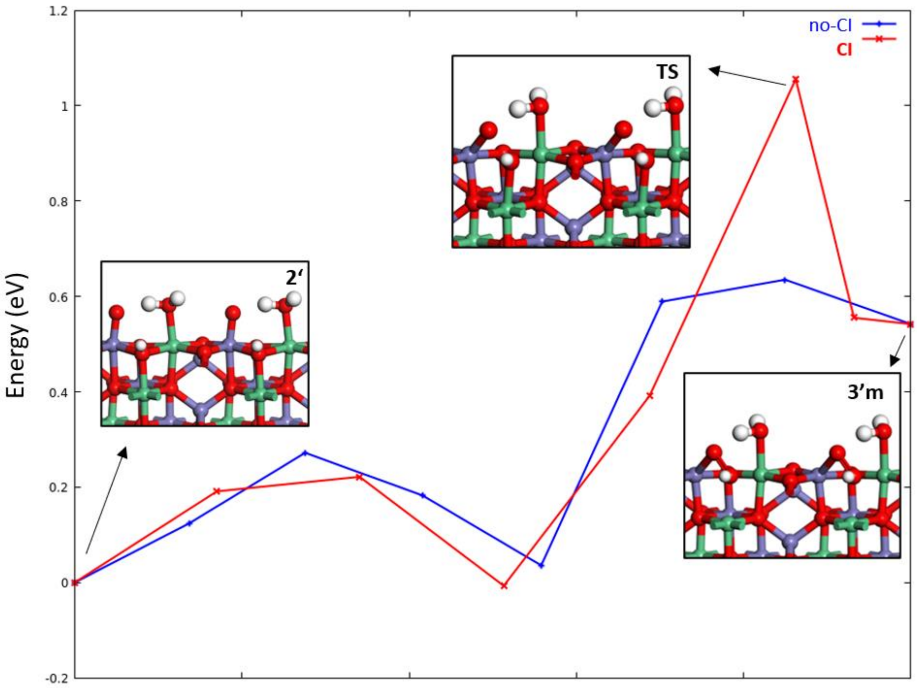

Figure S8. Nudge Elastic band (NEB) simulations for barrier search from state **2'** to **3m** (red line for CI, blue line for non-CI).

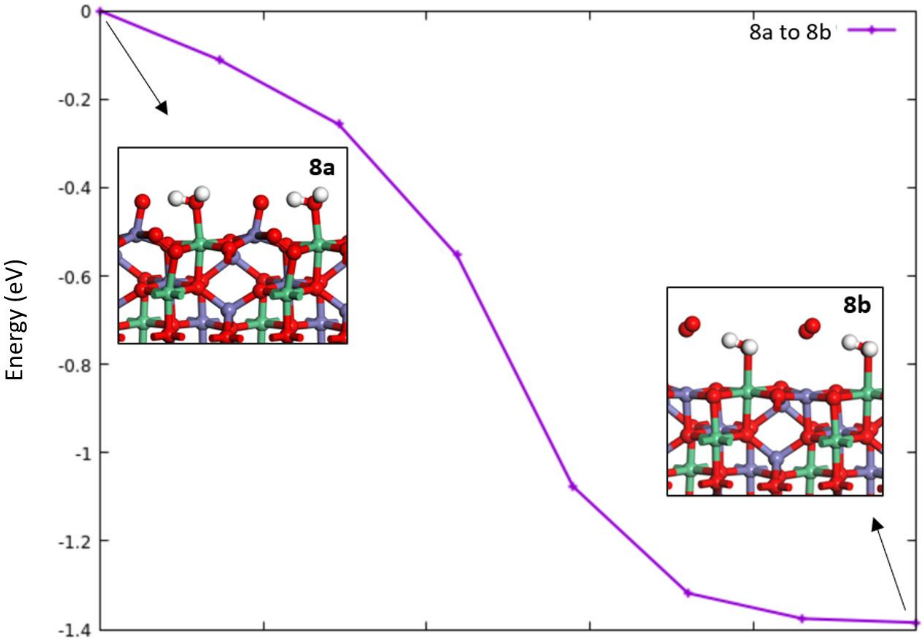

Figure S9. Nudge Elastic band (NEB) simulations for barrier search from **8a** to **8b.**

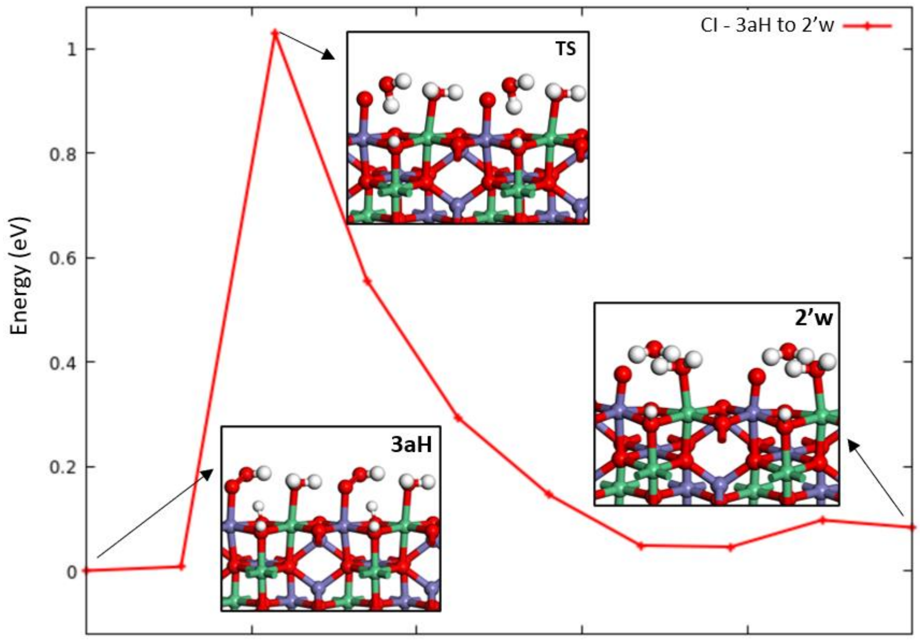

Figure S10. Nudge Elastic band (NEB) simulations for barrier search from **3aH** to **2'w** (CI).

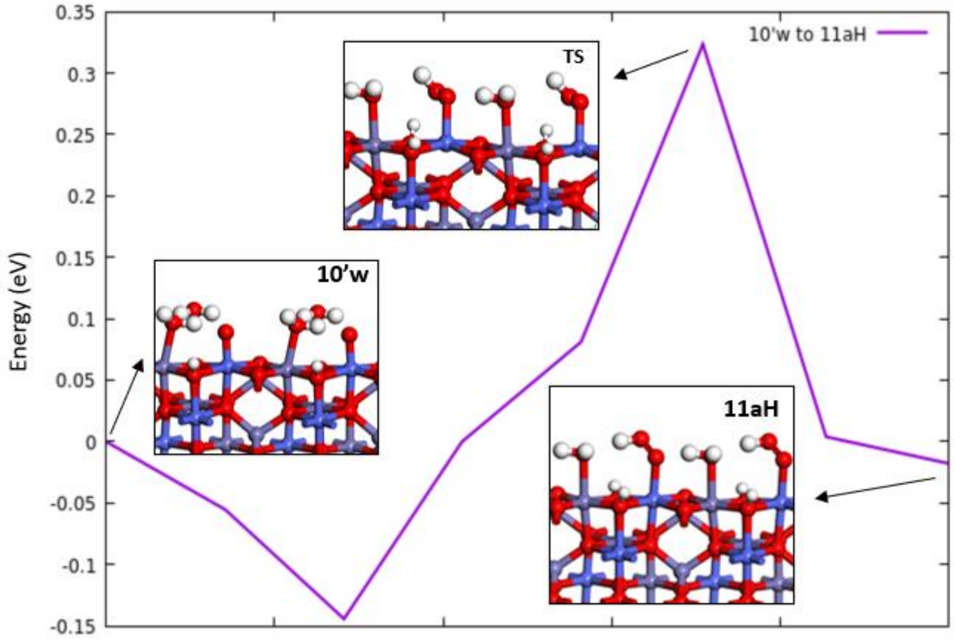

Figure S11. Nudge Elastic band (NEB) simulations for barrier search from **10'w** to **11aH.**

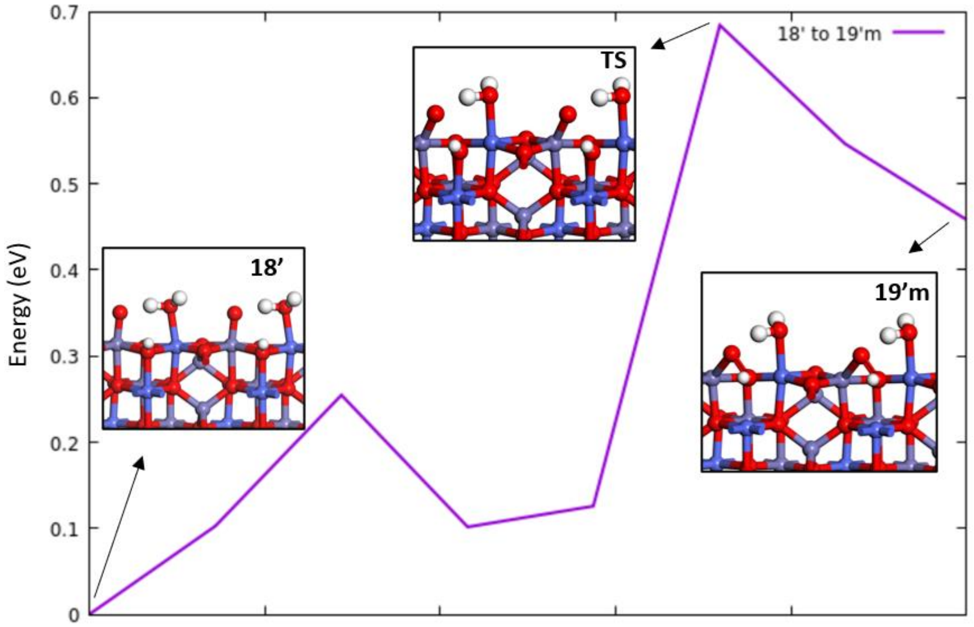

Figure S12. Nudge Elastic band (NEB) simulations for barrier search from **18’** to **19’m.**

3. Charged Calculations

Throughout the main text, we have investigated OER under acidic conditions. To investigate how the reaction mechanisms and their energetics may change in passing from acidic to alkaline conditions. we performed charged and implicit solvent calculations for one AEM step (**$*O + OH^- \rightarrow *OOH + e^-$**) of the OER reaction under alkaline conditions on $CoFe_2O_4$ to explore how this may change the mechanism and its energetics. Note that an AEM pathway is favored on $CoFe_2O_4$ when assisted at the Co site, so that this step (state **10'** to state **11a**) is the most likely candidate to explore role in the kinetics. Note also that this overall step occurs under acidic conditions as **($*O + H_2O \rightarrow *OOH + e^- + H^+$)** from state **10'** to state **11a** in (**Figure 9**) In detail, we have considered several distances at which OH^- is approaching the oxo oxygen in state 10'. We constrained only 2 atoms (oxygen of OH^-, and oxo oxygen) and kept the distance between these two atoms frozen, while relaxing the coordinates of all the other atoms at zero charge. We then used the Environ module[1] in the Quantum Espresso suite of codes on the geometries from the previous step to perform single point calculations in which a single negative charge of (-1) was added to the system, solvation was accounted for via the implicit-solvent model described in Ref. 1, and a charged plate was also added to mimic the electrochemical double layer, keeping the position of the charged plate fixed at ~3-4 Å above the top-most atoms in all the configurations, as illustrated in **Figure S13**. Finally, we performed a Löwdin population analysis of the Environ DFT wave functions to make sure that the OH species carries a negative charge, and indeed found a charge of ~ -6.7 *q* on the oxygen atom of OH in (a)-(d) in **Figure S13**.

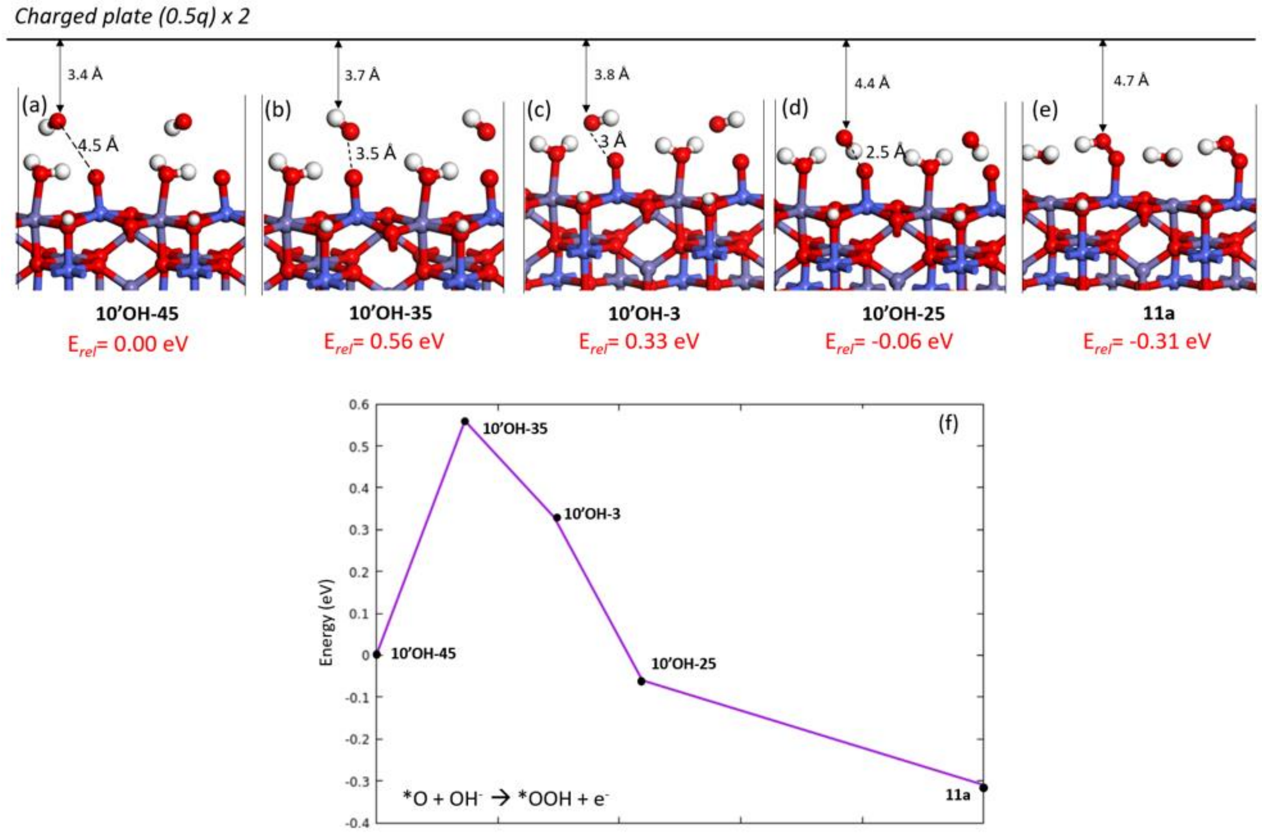

FigureS13. Configurations where OH^- is approaching the oxo oxygen in state **10'** at (a) 4.5 Å , (b) 3.5 Å, (c) 3 Å , (d) 2.5 Å , (e) state **11a**, solvent single point energies shown in red. (f) The energy profile of charged configurations.

As it can be seen in **Figure S13** (a)-(f), in this Eley-Rideal process of OH^-, an adsorption barrier[2] appears that we estimate to be 0.56 eV at an oxo/OH^- distance of about 3.5 Å. This barrier is due to de-solvation effects of the OH^- species. Since all the configurations are stoichiometric, in **Figure S13** we report the relative DFT/Environ single point energies.

Note that we did not find an overall barrier from state **10'** to state **11a** for the corresponding step under acidic scheme of **Figure 9**. The adsorption barrier is therefore an additional effect of charged systems. We emphasize that water adsorption (from **10'** to **10'w**) is indeed barrier-less under acidic conditions (**Figure 9**), while we estimate a barrier of 0.32 eV from state **10'w** to **11aH**, which is anyway smaller than the adsorption barrier of 0.56 eV in the OH^-- promoted path.

References

1. Andreussi, O. ; Dabo, I. ; Marzari. N. *J. Chem. Phys*. **2012**, 136, 064102.
2. Sementa, L.; Negreiros. F.; Fortunelli, A. 6 The use of hydrogen in ammonia synthesis, and in oxygen and carbon dioxide catalytic reduction – the reaction mechanisms. In: Van de Voorde M (ed.) *Volume 3 Utilization of Hydrogen for Sustainable Energy and Fuels*. Berlin, Boston: De Gruyter; **2021**. p.269-302.

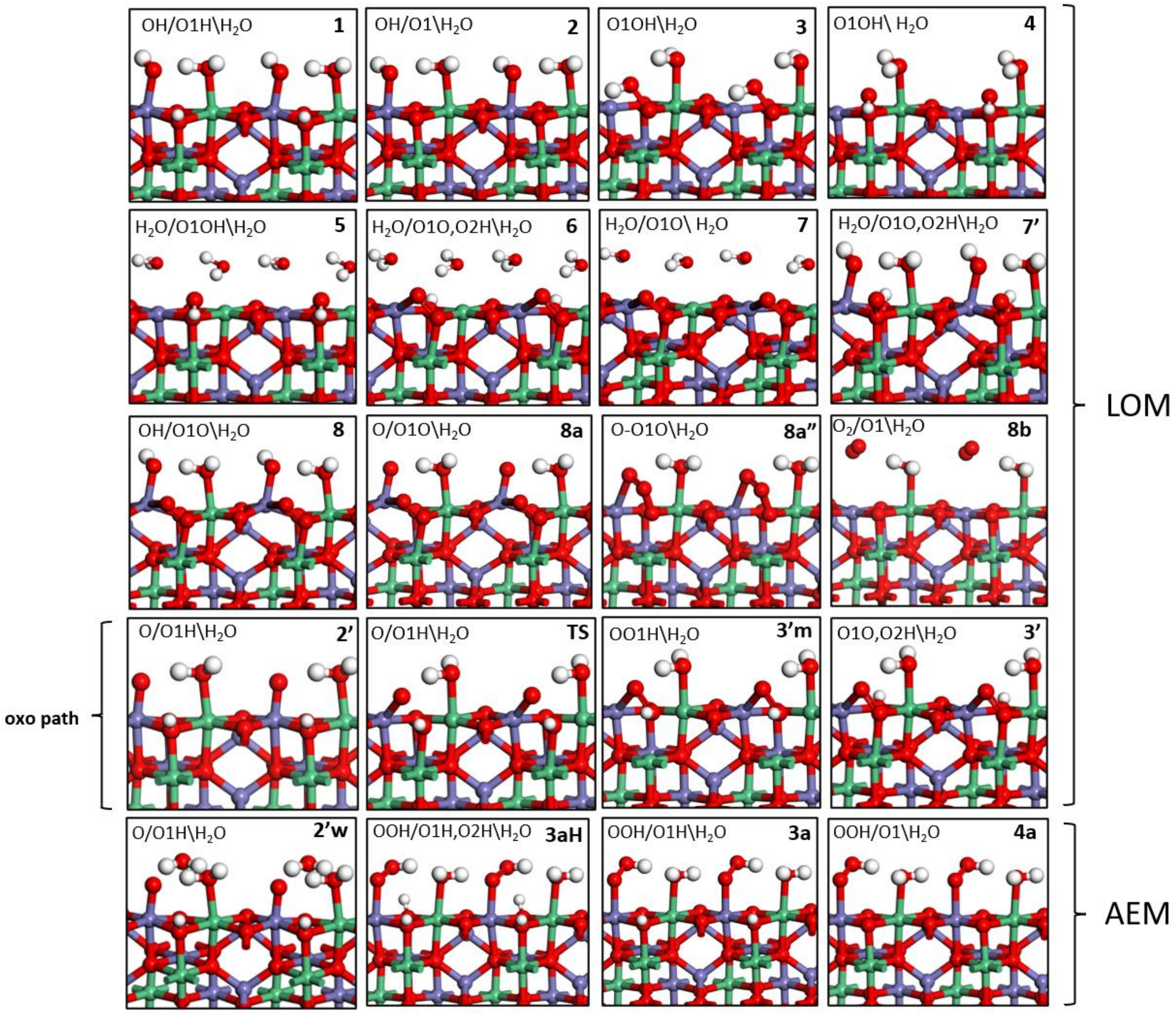

FigureS14. Optimized structures of the OER intermediates on (001) $NiFe_2O_4$. Oxygen, hydrogen, iron, and nickel atoms are colored red, white, violet, and green, respectively.

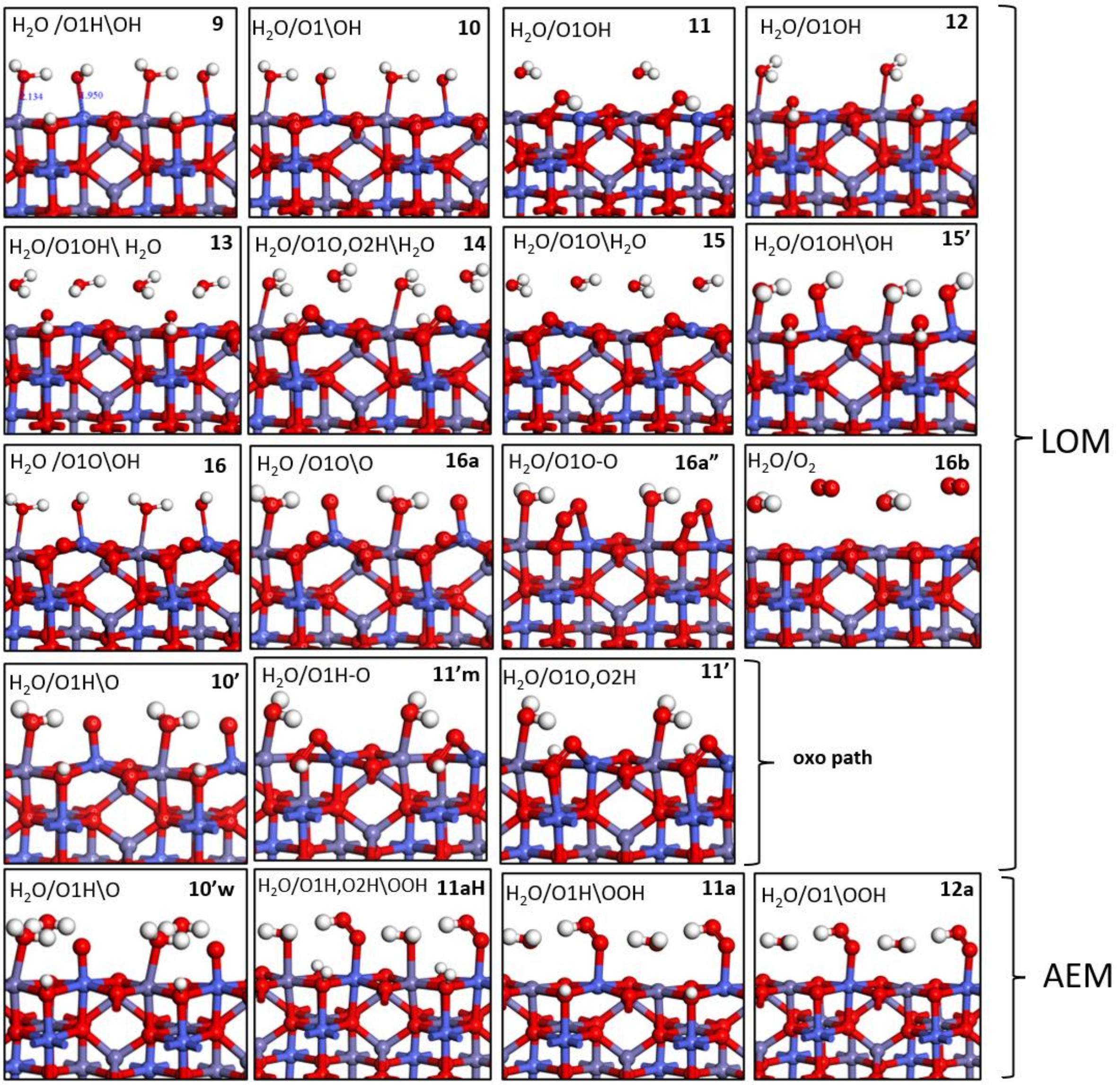

FigureS15. Optimized structures of the OER intermediates on (001) $CoFe_2O_4$ showing Co sites assist OER. Oxygen, hydrogen, iron, and nickel atoms are colored red, white, violet, and indigo-blue, respectively.

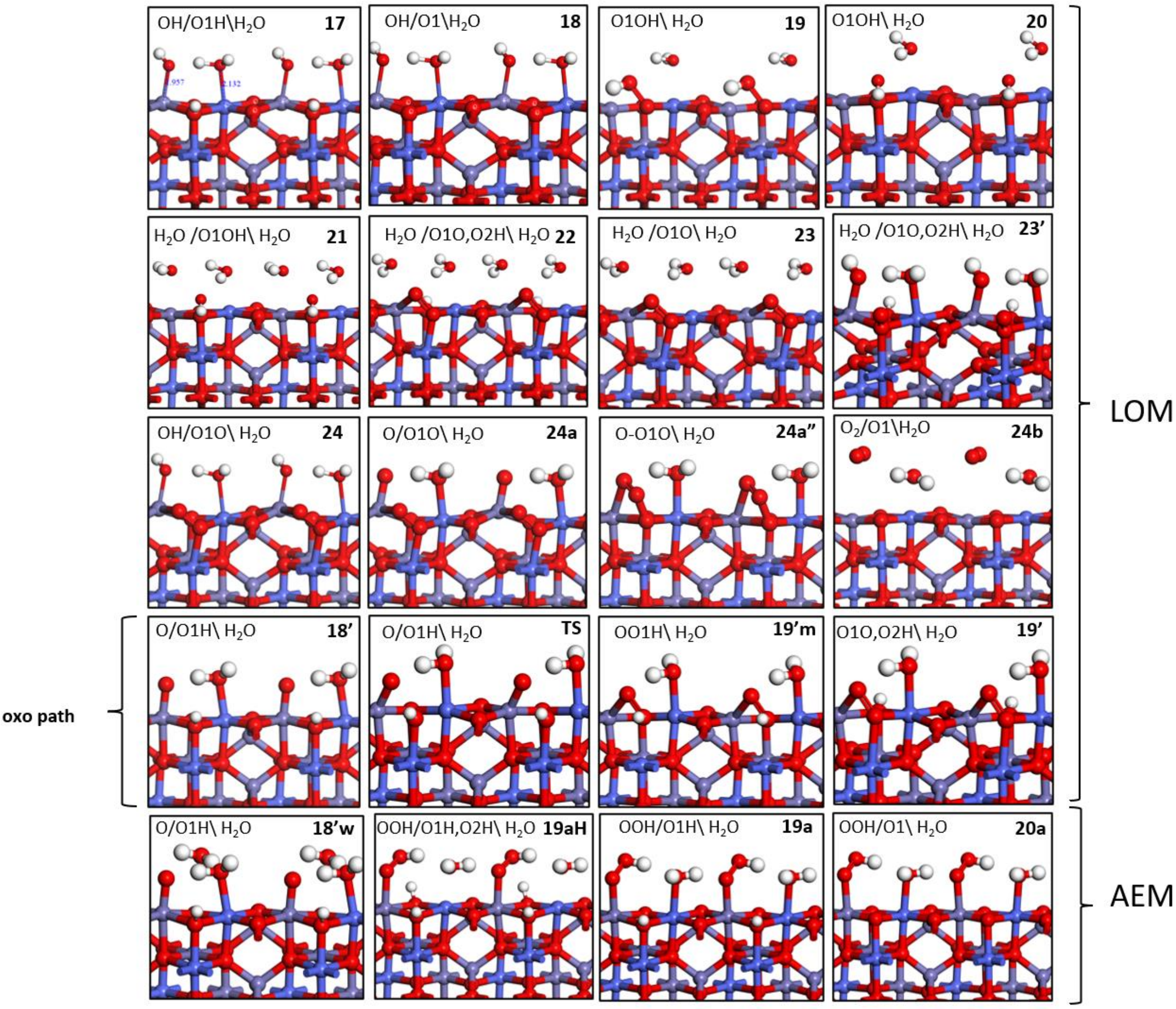

FigureS16. Optimized structures of the OER intermediates on (001) $CoFe_2O_4$ showing Fe sites assist OER. Oxygen, hydrogen, iron, and nickel atoms are colored red, white, violet, and indigo-blue, respectively.
